# Analyse multiple disease subtypes and build associated gene networks using genome-wide expression profiles

**DOI:** 10.1186/1471-2164-16-S5-S3

**Published:** 2015-05-26

**Authors:** Sara Aibar, Celia Fontanillo, Conrad Droste, Beatriz Roson-Burgo, Francisco J Campos-Laborie, Jesus M Hernandez-Rivas, Javier De Las Rivas

**Affiliations:** 1 Cancer Research Center (IMBCC, CSIC/USAL/IBSAL), Campus Miguel de Unamuno s/n, Salamanca, 37007, Spain; 2 Hematology Department, Hospital Universitario de Salamanca (HUS/IBSAL/USAL), Paseo San Vicente 58-182, Salamanca, 37007, Spain; 3 Celgene Institute for Translational Research Europe (CITRE), Parque Científico y Tecnológico Cartuja 93, c/Isaac Newton 4, Sevilla, 41092, Spain

**Keywords:** gene, expression, expression profile, gene networks, microarray, RNA-Seq, disease, disease classification, cancer, leukemia, acute leukemia

## Abstract

**Background:**

Despite the large increase of transcriptomic studies that look for gene signatures on diseases, there is still a need for integrative approaches that obtain separation of multiple pathological states providing robust selection of gene markers for each disease subtype and information about the possible links or relations between those genes.

**Results:**

We present a network-oriented and data-driven bioinformatic approach that searches for association of genes and diseases based on the analysis of genome-wide expression data derived from microarrays or RNA-Seq studies. The approach aims to **(i) **identify gene sets associated to different pathological states analysed together; **(ii) **identify a minimum subset within these genes that unequivocally differentiates and classifies the compared disease subtypes; **(iii) **provide a measurement of the *discriminant power *of these genes and **(iv) **identify links between the genes that characterise each of the disease subtypes. This bioinformatic approach is implemented in an R package, named *geNetClassifier*, available as an open access tool in Bioconductor. To illustrate the performance of the tool, we applied it to two independent datasets: 250 samples from patients with four major leukemia subtypes analysed using expression arrays; another leukemia dataset analysed with RNA-Seq that includes a subtype also present in the previous set. The results show the selection of key deregulated genes recently reported in the literature and assigned to the leukemia subtypes studied. We also show, using these independent datasets, the selection of similar genes in a network built for the same disease subtype.

**Conclusions:**

The construction of gene networks related to specific disease subtypes that include parameters such as gene-to-gene association, gene disease specificity and gene discriminant power can be very useful to draw gene-disease maps and to unravel the molecular features that characterize specific pathological states. The application of the bioinformatic tool here presented shows a neat way to achieve such molecular characterization of the diseases using genome-wide expression data.

## Background

Last decade of experimental work using genomic technologies has provided many data on gene expression profiling of different biological and pathological states [1]. This great effort in biomedical research has lead to a large need for tools and strategies that allow clinicians to translate the genome-wide expression data into useful information, such as transparent and robust signatures to characterize and distinguish multiple pathological subtypes [2]. There are many machine learning and computational procedures that can be applied to build classification systems that allow identifying the type or category of query samples whose class is not-known *a priori *[3-5]. However, a common problem of these methods is that they often do not reveal any information about the genes that are selected as variables for the classification process [4]. Although obtaining an efficient classifier might seem enough in some cases, there is a clear loss of biological information if the value or power of the chosen genes is not translated into parameters that allow to characterize and rank the genes.

Many clinical and biomedical studies look for the separation between multiple disease subtypes as distinct pathological states, but they are also very interested in finding the specific genes that are altered in each disease subtype. To identify and quantify the power of such 'marking genes' is the only way by which machine learning techniques can bring back biological meaning to this kind of biomedical studies. Moreover, gene products do not work in isolation as 'independent features', but rather interact with others in biomolecular networks to perform specific biological functions [6]. Therefore, together with the identification of the genes that mark a disease, genome-wide studies of related biological states should also provide information about the associations between the affected genes [7].

Following these questions we have developed a bioinformatic approach to provide gene-based analysis and characterization of different diseases and construction of associated gene networks using expression profiles derived from experimental transcriptomic data. The approach integrates established statistical and machine learning methods into a single tool that allows to **(i) **identify the set of genes that are specifically altered in a disease when a collection of several diseases (or disease subtypes) are studied and compared together using genome-wide expression profiling; **(ii) **obtain a minimum subset of these genes that enable to differentiate each disease subtype from the other; **(iii) **provide information about how relevant each of these genes is for discriminating each studied class; and **(iv) **find associations between the genes based on the analysis of the experimental expression profiles. This tool has been implemented in an R/Bioconductor package named *geNetClassifier *(available at http://www.bioconductor.org/). In order to validate the tool as a whole and prove whether the results it provides have biological and functional meaning, here we present its application to two independent genome-wide expression datasets of human samples isolated from individuals with different subtypes of leukemia: one using high-density oligonucleotide microarrays and another using deep RNA-sequencing.

## Results and discussion

### Finding genes associated to specific disease subtypes

**Figure 1:**
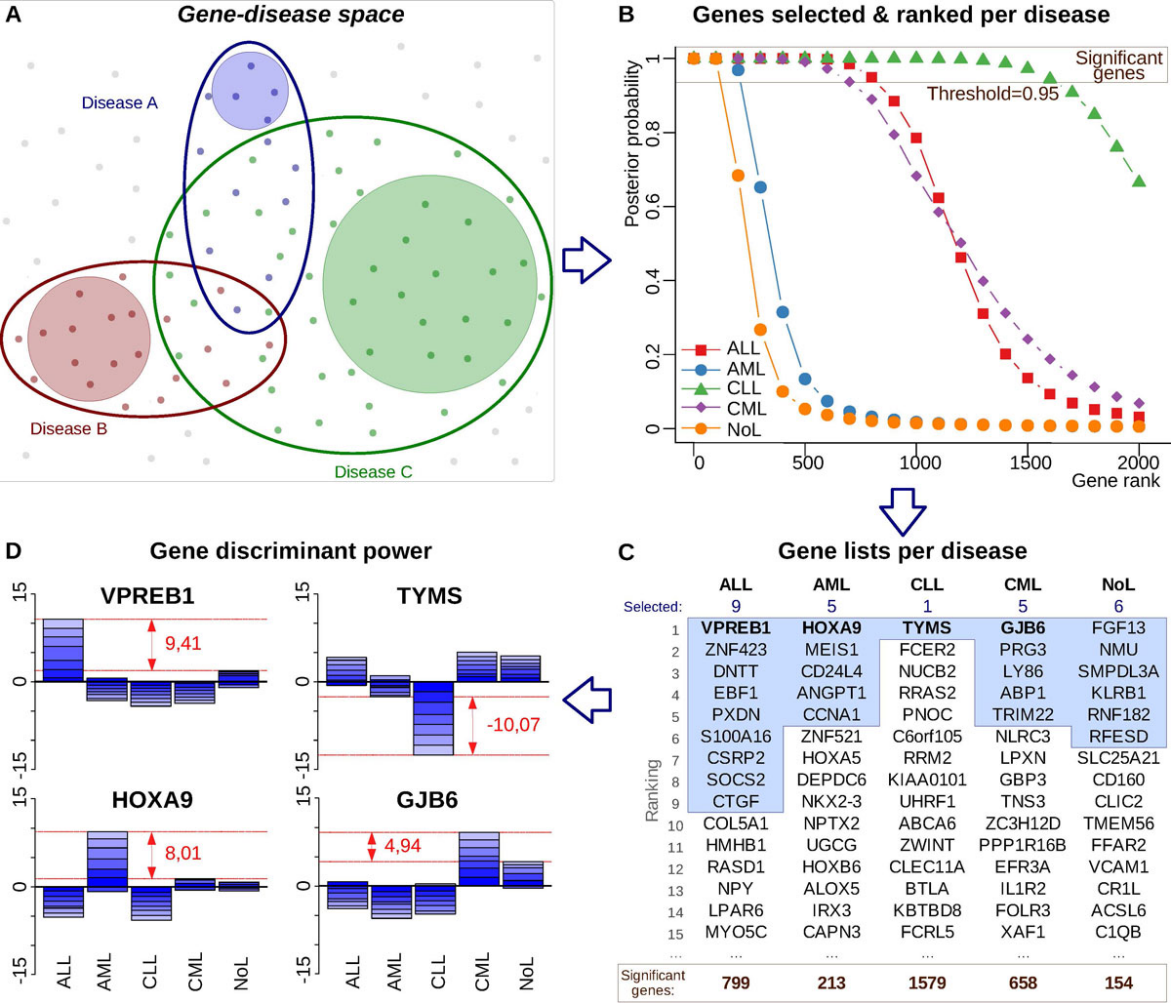
**Description of geNetClassifier main features and results**. **(A) **Scheme representing a gene-disease space for three hypothetical diseases. The color-line ovals would enclose the genes (dots) affected by a given disease. The coloured-background circles would mark the altered genes that are specific for a disease, which are the ones we aim to identify: i.e. genes affected by only one of the compared diseases. **(B) **Graph presenting the posterior probability of the top 2000 genes included in the gene ranking for each of the 4 leukemia subtypes and the non-leukemia samples. The genes are selected and ranked based on their posterior probability for each class. Genes with posterior probability over the threshold (> 0.95) can be considered *significant *candidates to mark each disease subtype. **(C) **Lists presenting the top-15 genes in the ranking of each class. The bottom row shows the total number of significant genes in the whole ranking. The shaded area contains the genes selected as the minimum subset to separate the classes. The number of genes selected per class is shown on top. **(D) **Discriminant power plot of the first ranked gene for each leukemia subtype: *VPREB1 *(pre-B lymphocyte 1) for ALL; *HOXA9 *(homeobox 9) for AML; *TYMS *(thymidylate synthetase) for CLL; and *GJB6 *(gap junction beta-6 30 kDa protein) for CML. The red numbers indicate the *discriminat power *values assigned to each gene.

The human gene landscape can be structured in functionally associated groups of genes which are specific to biological processes or states. Since a disease will normally affect and alter one or several biological processes, we could depict a theoretical multidimensional "gene space" divided in regions that include genes associated to specific pathological states (Figure 1A). The identification of these groups of genes is a great scientific endeavour for biomedical research, and some biological databases (e.g. OMIM [8]) have been built following the idea of a "gene-to-disease mapping", as it is known to happen in Mendelian inherited diseases. In this theoretical scenario, the genes that are affected by a given disease can be overlapping with the ones affected by a similar pathological state. This will define genes that can be altered in multiple pathologies, but it will also define genes that are only affected by a specific malignancy when compared with other diseases.

Considering the recognition of such theoretical gene-disease space (Figure 1A), we apply expression profiling to find the genes that are altered in one specific disease subtype using differential expression analysis. To do so, we compare each disease category versus all the others using package EBarrays [9], that implements an empirical Bayes method [10]. This provides a *posterior probability *for each gene to be differentially expressed in one of the classes (see Methods). Sorting the genes by their probability allows to build a ranking of the genes ordered by their statistical significance (Figure 1B). Since each gene has a probability of differential expession per class, it is assigned to the class in which it has the best ranking. This allows to build non-overlapping gene lists that optimize the specificity and separation between classes. The posterior probability also allows to quantify the association of a gene with a class and identify how many genes are related to each class at a certain significance level.

### Constructing gene-based classifiers for multiple diseases

Once the gene rankings have been established, the tool selects from the top of the list the minimum subset of genes required to identify each class. To achieve this, it uses a multiclass implementation [11] of Support Vector Machine (SVM), as a method that has been proven very efficient for classification of gene expression microarray datasets [12-14]. The SVM is integrated into a wrapper forward selection scheme to test whether a selected subset of genes is actually enough to discriminate the classes [15]. Several SVM classifiers are iterativelly trained with an increasing number of genes taken from the ranked lists and evaluated through double nested cross-validation. The smallest subset of genes that provides the best performance is selected as feature set (Figure 1C) and used to train and build a final classifier that will include all the available samples of the training set.

The classifier built for a given set of compared diseases can be used to query and identify new unlabeled samples. In addition, the classifier is analysed in order to obtain the *discriminant power *of the selected genes (Figure 1D). Each gene's *discriminant power *is a quantitative parameter that resembles the value of such gene in class differentiation. Therefore, a high *discriminant power *(either positive or negative, in absolute value) indicates that the gene is useful to mark and identify samples from its assigned class. Full description of this parameter is provided in Methods section.

### Building networks of genes associated to diseases

To infer possible associations between the genes assigned to each disease, *geNetClassifier *calculates gene-to-gene correlation and mutual information [16] in the expression dataset. This allows to identify possible relations of co-expression between the genes and possible relations of mutual redundancy. The detected associations are integrated in a network that also includes parameters derived from the differential expression analysis and from the classification analysis. Since networks are built for each class, they provide an integrative view of the gene sets associated to each disease in a relational characterized context. Examples of these networks are presented in the case studies in the following sections.

### Using *geNetClassifier*: analysis of a leukemia dataset

We have applied *geNetClassifier *to a dataset of genome-wide expression microarrays of samples from leukemia, as a well known disease that allows to test the tool in a real case study and confirm the biological relevance of the results. This dataset includes 50 microarray samples from bone marrow of patients of four major leukemia subtypes (ALL, AML, CLL and CML; described in Methods) plus non-leukemia controls (NoL), making a total of 5 distinct classes.

The first result that *geNetClassiffier *provides is the set of rank-ordered lists of genes selected for each class, being the top genes the ones most significantly associated with each disease (as indicated in Figure 1C). The resulting lists of genes-per-disease do not overlap, in this way the method is optimized to find specific markers of each compared disease. The number of genes associated to each disease for a common threshold of significance is quite different from one class to another (e.g. 799 genes for ALL but only 213 genes for AML). This observation seems to indicate that some diseases can affect more genes than others according to their comparative changes in the global expression profiles. These sizes do not represent the absolute number of genes each disease affects, but rather the genes that are only affected by each disease in the specific contrast. In any case, this phenomenological consideration supports the proposed hypothesis of a gene-disease space, where different diseases affect different number of genes.

After the classification process the minimun subset of genes that allow the best class separation were selected: 9 genes for ALL, 5 for AML, 1 for CLL, and 5 for CML (blue-shaded boxes in Figure 1C; detailed information about these genes is included in Additional File 1).

### External validation and performance of *geNetClassifier*

Once the classifier for leukemias was built, an external validation was conducted to evaluate the accuracy and performance of the algorithm and to confirm the robustness of the genes selected as markers of the corresponding classes [17].

**Figure 2:**
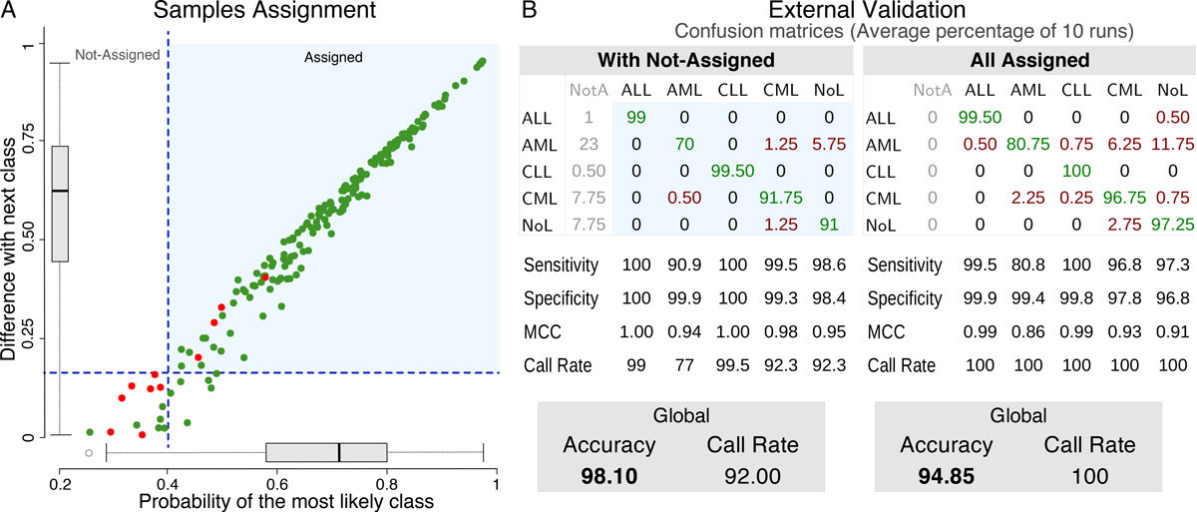
**External validation of geNetClassifier using a large dataset of leukemia samples**. **(A) **Assignment of 200 leukemia samples in one run of external validation. Green dots correspond to samples in which the most likely class is correct and red dots to samples in which it is incorrect. Samples in the blue background area are assigned to the most likely class; samples under any of the two thresholds (dashed blue lines) are considered doubtful samples and can be left as "Not-Assigned". **(B) **Summary of the results of 10 independent external validations. Average confusion matrices (in percentage values) and statistical parameters: sensitivity, specificity, MCC, call rate (calculated per class), global accuracy and global call rate (calculated globally for all the classes). On the right (labelled *All Assigned *) the statistics are calculated assigning all the samples to their most likely class. On the left (*With Not-Assigned *) the statistics are calculated leaving doubtful samples unassigned.

An external validation consists on querying the classification system with an independent set of samples whose class is *a priori *known. We used a different set of 200 samples of the same five classes (Figure 2). Sensitivity, specificity, MCC, global accuracy and global call rate were calculated to evaluate the performance. These statistical parameters were estimated in 10 runs of external validation randomly splitting the available samples.

The external validation could be performed following two different approaches: **(i) **assigning all the samples to their most likely class or **(ii)** leaving doubtful samples as *not-assigned*. (See Methods).

When the *not-assigned *option was selected, the external validation done with 200 leukemia samples provided an average of 4 misclassifications per run (shaded region in Figure 2A). All other samples were either correctly assigned or left unclassified (*not-assigned *), resulting in an average global accuracy of 98% and average call rate of 92% (assignment percentage). By contrast, since most samples that would have been incorrectly assigned had a probability under the thresholds (red dots in Figure 2A), the accuracy when all samples were forced to be assigned to their most likely class was 94.85%.

In overall, the external validation for the leukemias showed that the best performance (allowing not assignment) was obtained for ALL and CLL (100% sensitivity and specificity, MCC = 1.0), while nk-AML presented the lowest values (90.9% sensitivity, 0.94 MCC and 77% call rate). Difficulties in the identification and classification of nk-AMLs were already described in a large-scale international leukemia study where the rate of misclassification for this specific subtype was 11.4% [18]. In conclusion, the classification accuracy rates provided by *geNetClassifier *confirms that the genes sets selected for each class can be good markers of the analysed disease subtypes.

### Genes and networks associated to each leukemia subtype

**Figure 3:**
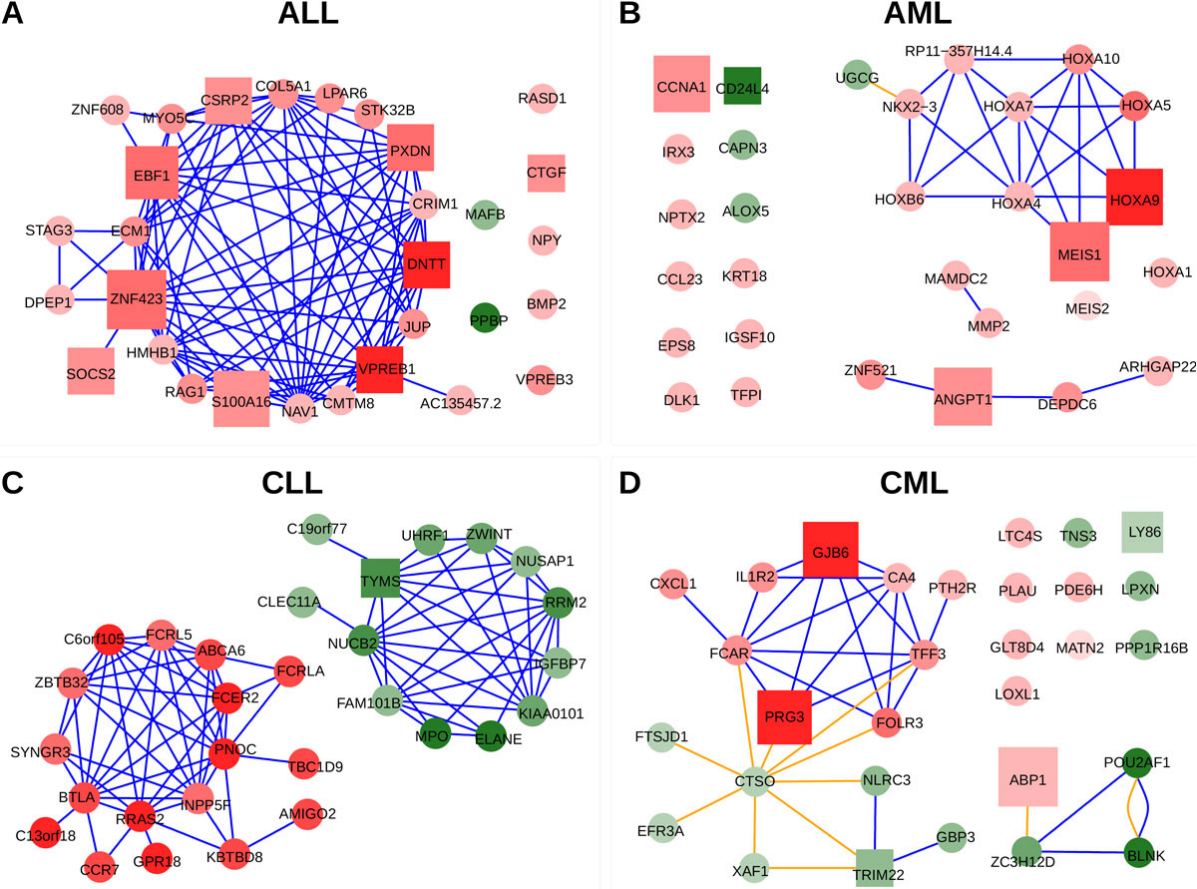
**Gene networks produced for each studied leukemia subtype**. The networks contain the top-30 ranked genes selected for each class. Genes are coloured in red if they are overexpressed and in green if they are repressed in the given subtype. The intensity of these colours indicate the magnitude of the observed expression difference. Squares correspond to genes that were selected as *minimum subset *to differentiate the classes. The size of these squares is proportional to the *discriminant power *of the gene. The color of the edges indicates the type of relations between the genes: blue meaning co-expression (correlation) and orange mutual information.

The gene networks produced for each leukemia subtype are presented in Figure 3. The plots include the top-30 genes selected for each class as characteristic markers of each leukemia subtype.

Several of these genes have been already reported as functionally associated to these diseases. For example, in the case of ALL, the gene *VPREB1 *-that is the first gene in ALL ranking-encodes a protein that belongs to the immunoglobulin superfamily and is expressed selectively at the early stages of B lymphocytes development (i.e. on the surface of pro-B and early pre-B cells). This gene has already been proposed as a useful marker for the detection of normal and malignant human pre-B lymphocytes [19]. Since all ALL samples included in this study correspond to pre-B-ALL without t(9;22), the selection of *VPREB1 *seems quite adequate. Another gene selected to mark ALL is *DNTT*. The protein encoded by *DNTT *is expressed in a restricted population of normal and malignant pre-B and pre-T lymphocytes during early differentiation.

In the case of the genes selected for nk-AML, the network shows a cluster of homeobox genes (*HOXA4*, *HOXA5*, *HOXA7*, *HOXA9*, *HOXA10*). The co-expression of these genes detected in the dataset reveals that they are coregulated. *MEIS1 *is a transcriptional regulator also included in the homeobox co-expression cluster and selected as one of the genes with best discriminant power for the nk-AML class. Two recent publications have reported that downregulation of *MEIS1 *and *HOXA *genes impair proliferation and expansion of acute myeloid leukemia cells [20,21]. Moreover, *HOXA *has a specific translocation event that has been associated with myeloid leukemogenesis, and overexpression of *HOXA9 *has been shown as representitative of nk-AML patients during first diagnosis and if they suffer relapse [22]. These and other reports support the selection of *MEIS1 *and *HOXA9 *in the gene network that characterizes AML with normal karyotype [23]. Another gene related to AML is *ANGPT1*, that encodes protein angiopoietin 1. Angiopoietins are proteins with important roles in vascular development and angiogenesis which have also been identified as over expressed in bone marrow of AML patients [24].

Finally, the gene network produced for CML includes characteristic genes such as *PRG3*, that encodes for eosinophil major basic protein 2 (MBP2) which is specific of eosinophil granulocytes, a myeloid cell type. Moreover, it has been shown that many molecules essential for tumor cell growth (like polyamines) enter cells via a proteoglycan-dependent pathway that involves PRG3 [25]. All these published reports do not prove that the genes included in the networks for each leukemia subtype are essential for the development of such diseases. However, they give important support to the results and underline the value of the method for creating significant gene sets and gene networks associated to specific disease subtypes.

### Application of *geNetClassifier *to an RNA-Seq dataset

*geNetClassifier *can be applied to different types of genomic data produced with different platforms. We have also applied it to an RNA-Seq dataset of acute leukemia samples [26] from which we selected 45 samples from patients with two AML subtypes: **(i) **11 samples of patients with t(15;17) chromosomal translocation characteristic of acute promyelocytic leukemia (APL), and **(ii) **34 samples of AML patients with normal karyotype and no detected FISH abnormalities (nk-AML). APL is an AML subtype that has good clinical prognosis. Its sensitivity to all-trans retinoic acid (ATRA) allows an efficient treatment unique among leukemias. By contrast, nk-AML is one of the most frequent subtypes of AML (approx. 50%) and usually has a poor clinical prognosis due to the lack of an efficient treatment [26]. Out of these two AML subtypes, nk-AML was also present in the previous microarray dataset analysed. This allows us to investigate the performance of the algorithm studying a common disease subtype in a different context and using a different type of expression data.

**Figure 4:**
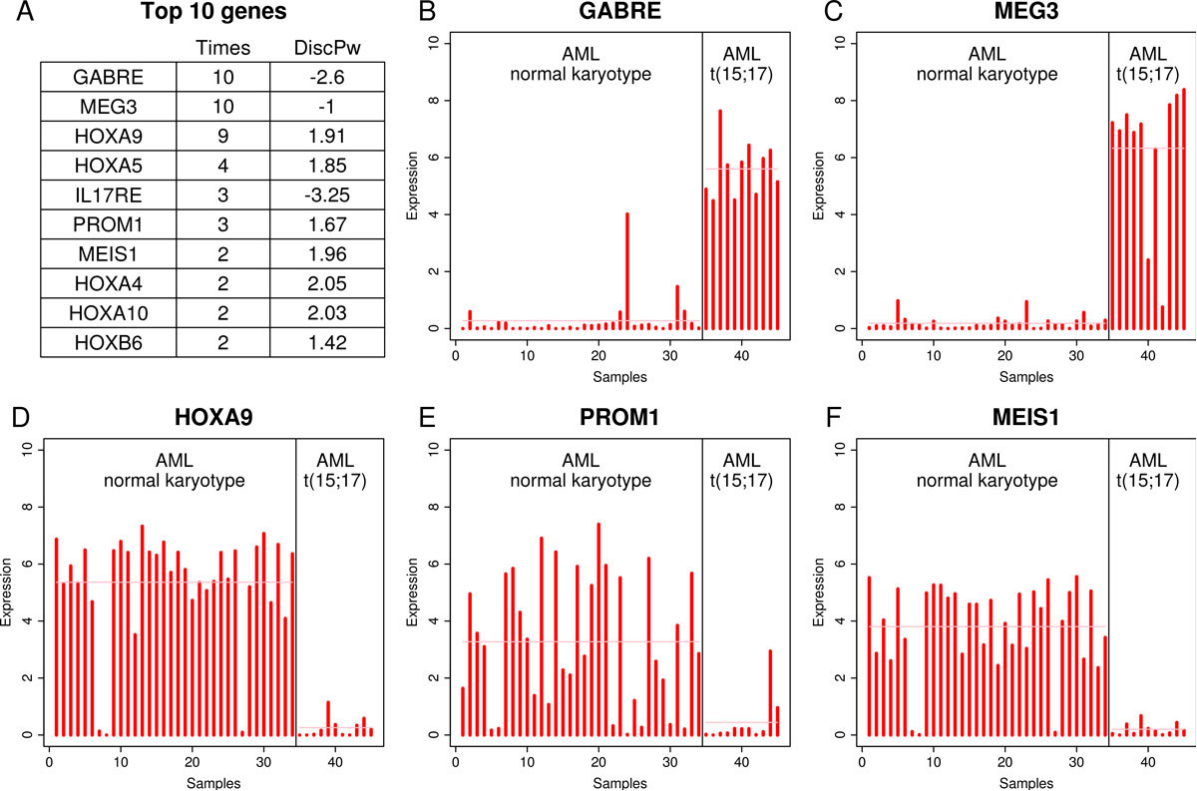
**Genes selected for the RNA-seq dataset of acute leukemia**. **(A) **The table shows the genes most frequently selected to distinguish between normal karyoptype AML (nk-AML) and AML with t(15;17) (APL). *geNetClassifier *was run ten times with different combinations of samples. The table includes the mean discriminant power within these runs and the number of times that each gene is included in the *minimum subset*. **(B-F) **Expression profiles corresponding to some of the top selected genes: *GABRE*, *MEG3*, *HOXA9*, *PROM1 *and *MEIS1*. Each red bar corresponds to the expression signal *(log_2_(RPKM+1))* in one sample.

**Figure 5:**
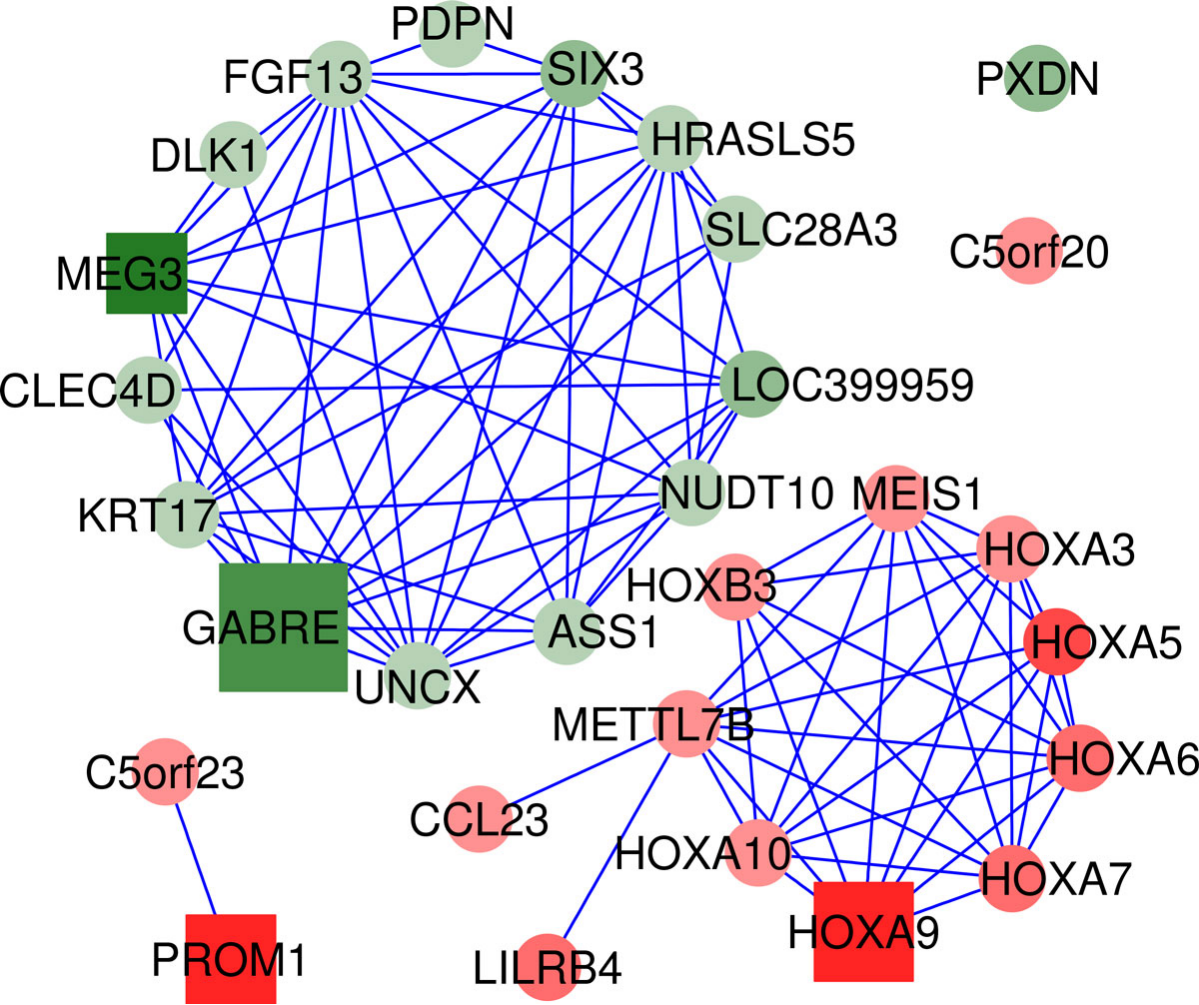
**Gene network obtained for AML with the RNA-seq dataset**. The network contains the top-30 ranked genes selected after running *geNetClasifier *to analyse the RNA-Seq expression data of normal karyoptype AML (nk-AML) versus AML with t(15;17) (APL) samples. The network shows two clear clusters: one including genes that are up-regulated in nk-AML and another with down-regulated genes. The red cluster includes many homeobox (HOX) genes highly correlated. These genes are characteristic of nk-AMLs and show good agreement with the results obtained with microarrays in spite of being two totally independent datasets.

*geNetClassifier *was applied to the RNA-Seq dataset of APLs and nk-AMLs using 8 samples from each class as training samples and then validated with the rest of the samples. We repeated this process 10 times randomly selecting the training samples. The global accuracy obtained in this analysis was 100% with a call rate of 91.38%. The list of genes most frequently selected for classification (Figure 4A) included several homeobox genes (HOXA and HOXB) and MEIS1, showing agreement with the results obtained for nk-AML in the microarray analysis. In this way, the expression profiles from these genes in the RNA-Seq dataset are consistent with the results obtained with the array dataset, e.g.: genes *HOXA9 *and *MEIS1 *were down regulated in APL in comparison to nk-AML (Figure 4D and 4F). In addition, the network generated for nk-AML selected a set of homeobox genes that form a highly connected co-expression cluster (Figure 5). Other genes detected in this analysis, for example MEG3, showed over-expression in APL versus nk-AML (Figure 4C). In fact, it has been reported that MEG3 expression is lost in multiple cancer cell lines of various tissue origins and it inhibits tumor cell proliferation in vitro. The identification of MEG3 as marker over-expressed in the AML subtype with better prognosis (i.e. APL) provides support to the selection of this gene as a discriminant feature between APL and nk-AML.

Finally, to have a better estimation of the global agreement provided by the algorithm in the analysis of the genes assigned to a given disease subtype, we analysed the total overlapping of the genes selected for nk-AML in the arrays dataset and the RNA-Seq dataset. Both platforms included a common set of 16,611 human protein-coding genes. Within this set, the number of significant genes selected for nk-AML were 202 (using posterior probability > 0.95). The RNA-Seq results included 95 of these genes (considering the 10 runs indicated above), and 76 of them were selected in more than three runs. An overlap of 95 genes corresponds to an odds ratio of 3.27 and to an enrichment p-value < 0.000001 (using hypergeometric test). Therefore, it can be said that the consistency of the method to select genes that mark a specific disease subtype is high.

### Comparison of *geNetClassifier *with other methods

Finally, we have evaluated the performance of *geNetClassifier *relative to other gene selection and classification methodologies. We compared *geNetClassifier *with four machine learning methods for feature selection using CMA package [27], which provides a comprehensive collection of various microarray-based classification algorithms (see Additional File 2). We have also evaluated the classification procedure of *geNetClassifier *using svb-IMPROVER contest platform [28], which includes a Diagnostic Signature Challenge with several datasets to assess and verify computational approaches that classify clinical samples based on transcriptomics data (see Additional File 3). In both cases, the performance of *geNetClassifier *algorithm is within the best methods. However, it should be noted that we could only compare the classification and gene selection procedures. The other features included in our package could not be found integrated in other methods.

## Conclusions

Biological annotation of the genes selected and the networks built to mark and separate different pathological states confirm the value of using *geNetClassifier *to analyse multiple disease subtypes based on genome-wide expression profiles. The tool is provided open access in Bioconductor to facilitate the type of studies illustrated in this report.

As a general conclusion, the results using *geNetClassifier *showed a robust selection of gene markers for characterizing disease subtypes and allowed the construction of specific and weighted gene networks associated to each disease subtype. The method can be applied to data derived from different types of technologies (such as microarrays or RNA-Seq) and it is designed to analyse datasets with multiple categories of samples.

## Methods

### Implementation and availability

*geNetClassifier *has been developed as an R package following Bioconductor (BioC) standards and technical requisites (http://www.bioconductor.org). It has attained BioC package submission process and package guidelines to be included in BioC software release. It is freely available, open source and open access. The package includes help pages with usage examples for each specific function. Together with the package, we have written a *vignette *including a detailed tutorial to use the algorithm (Additional File 4).

### Microarray dataset

The microarray leukemia dataset is a subset of 250 samples collected from the Microarray Innovations in Leukemia (MILE) study [18] available at Gene Expression Omnibus database (http://www.ncbi.nlm.nih.gov/geo/) under series accession number GSE13159. The genome-wide expression signal corresponding to these samples was measured using *Affymetrix *Human Genome U133 Plus 2.0 microarrays. The samples correspond to mononuclear cells isolated by Ficoll density centrifugation from bone marrow of untreated patients with: **(1) ***Acute Lymphoblastic Leukemia *(ALL) subtype *childhood *or *precursor B-cell *(c-ALL/pre-B-ALL) without translocation t(9;22); **(2) ***Acute Myeloid Leukemia *(AML) subtype *normal karyotype *(nk); **(3) ***Chronic Lymphocytic Leukemia *(CLL) subtype *B-cell *; **(4) ***Chronic Myeloid Leukemia *(CML); **(5) ***Non-leukemia *and healthy bone marrow (NoL).

The microarrays were normalized using the algorithm Robust Multi-Array Average (RMA) [29] and applying a gene-centric redefinition of the probes from the *Affymetrix *arrays to Ensembl genes (Ensembl IDs ENSG). This alternative Chip Definition File (CDF) with complete unambiguous mapping of microarray probes to genes is available at GATExplorer (http://bioinfow.dep.usal.es/xgate/) [30].

### RNA-Seq dataset

The leukemia dataset analysed with RNA-sequencing corresponds to a subset of samples collected by the Cancer Genome Atlas (TCGA) [26] available at the TCGA data portal (https://tcga-data.nci.nih.gov/). These RNA-Seq data correspond to samples obtained from bone marrow aspirate of patients with AMLs of *de novo *diagnosis. Out of the available samples in TCGA, we selected 45 samples of the following subtypes: **(1) **AML patients with translocation t(15;17) (also called *Acute Promyelocytic Leukemia*, APL) (11 samples); and **(2) **AML patients with normal karyotype and no detected FISH abnormalities (nk-AML) (34 samples). The preprocessed RNA-Seq expression data matrices containing the *reads per kilobase per million mapped reads *(RPKM) were downloaded from the TCGA data portal and were log2 transformed *(log2(RPKM+1))* prior to be analysed with *geNetClassifier*.

### Statistical methods and algorithm procedures

#### Gene ranking

To create the gene ranking, *geNetClassifier *uses the function *emfit*, a Parametric Empirical Bayes method, included in package *EBarrays *[9]. This method implements an expectation-maximization (EM) algorithm for gene expression mixture models, which compares the patterns of differential expression across multiple conditions and provides a *posterior probability*. The posterior probability is calculated for each gene-class pair with a *One-versus-Rest *contrast: comparing the samples of one class *versus *all the other samples. In this way, the posterior probability represents how much each gene differentiates a class from the other classes (being 1 the best value, and 0 the worst). The ranking is built, in a first step, by ordering the genes decreasingly by their posterior probability for each class. To resolve ties, the algorithm uses the value of the difference between the signal expression mean for each gene in the given class and the mean in the closest class. In a second step, the ranking procedure assigns each gene to the class in which it has the best ranking. As a result of this process, even if a gene is found associated to several classes during the expression analysis, it will only be on the ranking of its best class. In addition, genes that do not show any significant difference between classes are filtered out before building the ranking. Finally, the set of genes considered *significant *in the ranking of each class is determined by a threshold of the posterior probability, which by default is set up to be greater than 0.95.

#### Classifier

The classifier included in the algorithm is a multi-class *Support Vector Machine *(SVM) available in R package *e1071 *[11]. This package provides a linear kernel implementation that allows the classification of multiple classes by using a *One-versus-One *(OvO) approach, in which all the binary classifications are fitted and the correct class is found based on a voting system.

#### Gene selection

The gene selection is done through a *wrapper forward *selection scheme based on *8-fold cross-validation*. Each cross-validation iteration starts with the first ranked gene of each class: it trains a temporary internal classifier with these genes, and evaluates its performance. One more gene is added in each step to those classes for which a 'perfect prediction' is not achieved (i.e. in case not all samples are correctly identified). The genes are taken in order from the *gene ranking *of each class until reaching zero error or the maximum number of genes allowed (determined by the arguments *maxGenesTrain *and *continueZeroError *). The error for each of the classifiers and the number of genes used to construct them are saved. Once the cross-validation loop is finished, it selects the minimum number of genes per class which produced the classifier with minimum error. To achieve the best stability in the number of selected genes, the cross-validation is repeated with new samplings as many times as indicated by the user (6 times by default). In each of these iterations, the minor number of genes that provided the smallest error is selected. The final selection is done based on the genes selected in each of the iterations. For each class, the top ranked genes are selected by taking the 'highest number' of genes selected in the cross-validaton iterations, but excluding possible 'outlier numbers' (i.e. selecting trimmed values).

#### Discriminant power

The *discriminant power *is a parameter calculated based on the *Lagrange coefficients *(alpha) of the *support vectors *for all the genes selected for the classification. Since the multi-class SVM algorithm is a *One-versus-One *implementation, it produces a set of *support vectors *for each binary comparison between classes. For each gene, the *Lagrange coefficients *of all the *support vectors *for each class are added up to give a value per class (represented as piled up bars in Figure 1D). The *discriminant power *is then calculated as the difference between the largest value and the closest one (i.e. the distance marked by two red lines in the plots in Figure 1D).

#### Assignment conditions

The whole tool *geNetClassifier *is built considering an *expert decision system *approach, because once the classifier is build it keeps open the possibility of '*do not assign*' when it is not sure about the class of a query sample. To make the assignment decision the probability to assign a sample to a given class should be at least double than the *random probability*, and the difference with the second most likely class should be higher than 0.8 times the *random probability*. If these conditions are not met, the sample is left as *Not-Assigned *(NA). These probability thresholds for assignment conditions are set up by default, but they can be changed by the user.

## List of abbreviations used

MCC: Matthews Correlation Coefficient

t(9;22): translocation between chromosomes 9 and 22

## Competing interests

The authors declare that they have no competing interests. Since performing the work described, CF has become an employee of Celgene Research S.L., part of the Celgene Corporation. The work here presented was done while CF was working at the Cancer Research Center (IMBCC, CSIC/USAL).

## Authors' contributions

SA carried out the development of the tool, performed its validation, carried out the implementation of the R package and drafted the manuscript. CF participated in the design of the study, carried out the development of the algorithm and the trials of the methods included. CD participated in the application of the package. BR participated in the validation of the methods and helped in writing the manuscript. FJCL participated in the validation and application of the algorithm with different datasets. JMHR participated in the selection and analyses of the diseases and provided the clinical patient samples. JDLR conceived the study, directed the design and development of the algorithm and wrote the manuscript. All authors read and approved the final manuscript.

## Supplementary Material

Additional file 1 **Table S1**. Table with data and information about the genes selected by *geNetClassifier *in the analyses of the leukemia microarrays dataset (classes: four leukemia subtypes and control class NoL): **Class**: The category a gene has been assigned to. **Rank**: Position of the gene within the list of genes ranked by significance assigned to a disease. **Posterior probability**: Probability value given by the expectation-maximization algorithm to each gene. This value is used to establish the ranking. In this result all values were very close to 1 (with more than 10 significant digits). Ties are further ranked based on the differential expression. **Expression**: Difference between the mean expression of the gene within its class and the mean expression in the other classes. UP or DOWN indicates whether the gene is overexpressed or repressed in its class compared to the other classes. **Discriminant Power**: Parameter calculated based on the *Lagrange *coefficients of the support vectors of the classifier. Represents the weight that the classifier gives to each gene to differentiate a given class. **Redundancy**: If TRUE, the gene has a high correlation or mutual information with other genes in the list. The threshold to consider a gene redundant can be set through the arguments (by default: correlations Threshold = 0.8 and interactions Threshold = 0.5). **Chosen for classification**: Number of times the gene was chosen for classification (as part of the minimum required subset) in the 5 internal cross-validation loops. Rank mean and rank standard deviation (SD) of the gene in these classifiers. **Cross-validation**: Mean and standard deviation of the rank that the gene has obtained in *geNetClassifier*'s internal cross-validation, including the times it was not selected for classification. Click here for file

Additional file 2 **Table S2**. Comparison of *geNetClassifier *gene selection procedure with four other machine learning methods for gene selection (i.e. feature selection): Limma, F-test, Boosting and Random Forest. The comparison has been done on the dataset of 250 leukemia samples, using R/Bioc package CMA that provides a comprehensive collection of various microarray-based classification algorithms [27]. Click here for file

Additional file 3 **File S3**. Evaluation of the performance of *geNetClassifier *classification procedure in the sbv-IMPROVER contest platform (https://sbvimprover.com/), which includes a Diagnostic Signature Challenge to assess and verify computational approaches that classify clinical samples based on transcriptomics data [28]. The performance has been evaluated using the dataset from IMPROVER that includes four classes corresponding to lung cancer subtypes. Click here for file

Additional file 4 **File S4**. *geNetClassifier vignette *including a tutorial with executable examples and description of all the methods. This *vignette *is available in Bioconductor: http://www.bioconductor.org/packages/release/bioc/vignettes/geNetClassifier/inst/doc/geNetClassifier-vignette.pdf Click here for file

## Declarations

The publication costs for this article were funded by the National Institute of Health Carlos III (ISCiii, Madrid, Spain) through grant PI12/00624.

This article has been published as part of *BMC Genomics *Volume 16 Supplement 5, 2015: Proceedings of the 10th International Conference of the Brazilian Association for Bioinformatics and Computational Biology (X-Meeting 2014). The full contents of the supplement are available online at http://www.biomedcentral.com/bmcgenomics/supplements/16/S5.
