# Supplementary material for: Analyse multiple disease subtypes and build associated gene networks using genome-wide expression profiles
Source: BMC Genomics. 2015 May 26;16(Suppl 5):S3. doi: 10.1186/1471-2164-16-S5-S3 (PMC4460584; doi:10.1186/1471-2164-16-S5-S3)
Supplement: Additional file 3 — File S3. Evaluation of the performance of geNetClassifier classification procedure in the sbv-IMPROVER contest platform (https://sbvimprover.com/), which includes a Diagnostic Signature Challenge to assess and verify computational approaches that classify clinical samples based on transcriptomics data [28]. The performance has been evaluated using the dataset from IMPROVER that includes four classes corresponding to lung cancer subtypes. [file 1471-2164-16-S5-S3-S3.pdf]

Additional file 3: File S3.

Evaluation of the performance of ***geNetClassifier*** classification procedure in the **sbv-IMPROVER** contest platform (https://sbvimprover.com/challenge-1), which includes a **Diagnostic Signature Challenge** to assess and verify computational approaches that classify clinical samples based on transcriptomics data.

The performance of the algorithm ***geNetClassifier*** has been evaluated using a data-set that has multiple classes (a data-set of **lung cancer** included in **IMPROVER**). We show below the results corresponding to the performance measured with three parameters: **(i) AUPR**, that computes the precision-recall curve for each class, from which the Area Under the Precision-Recall curve is extracted (Precision is a measure of specificity whereas Recall is a measure of completeness); **(ii) BCM**, Belief Confusion Matrix, that is a matrix whose element {i,j} is the average confidence that a sample belonging to class i is in class j (Each prediction has its own belief confusion matrix. The perfect belief confusion matrix is the identity matrix); **(iii) CCEM**, Correct Class Enrichment Metric, that is computed adding the confidence of the samples whose classes were correctly predicted and subtract the confidence of the subjects whose classes were incorrectly predicted (In other words, this is a measure of enrichment of the correctly classified samples. The final value is normalized to be between 0 and 1).

These parameters indicate, as shown in the tables below, that ***geNetClassifier*** is within the best methods, performing as the **6th best** out of 47 different methods submitted to the **Diagnostic Signature Challenge** when it is applied using the option of **"not-assignment"**; and as the **7th best** in the rank of 47 methods when it is used forced to assign always a query sample to a class (**"all assigned"**).

Plots that present the results of AUPR, BCM and CCEM corresponding to the performance of ***geNetClassifier*** using the option of **"not-assignment"**.

The RESULTS TABLE placed after these plots presents the values of these parameters and the rank for the top-15 methods.

AUPR

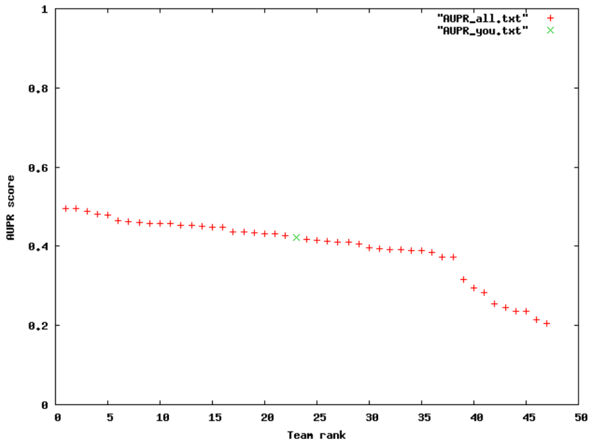

BCM

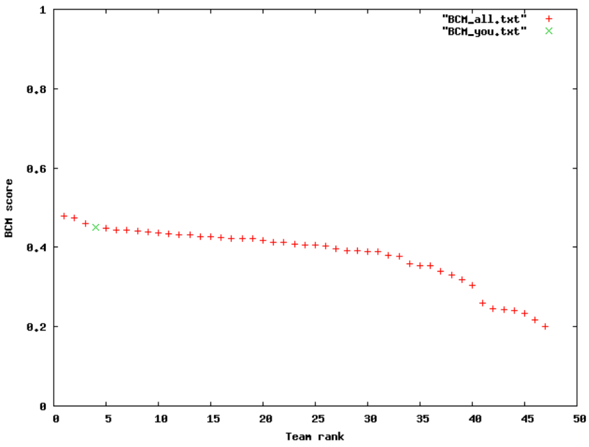

CCEM

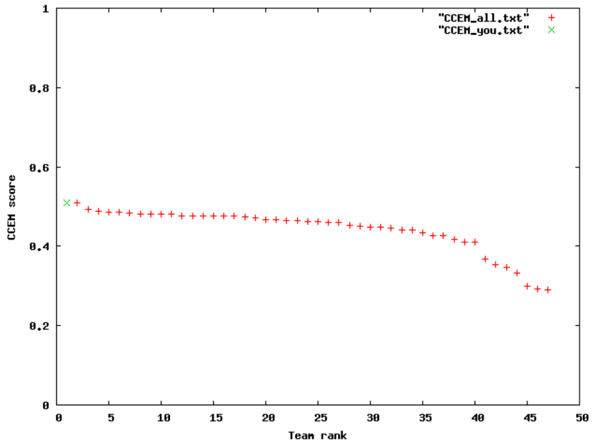

RESULTS TABLE

Team	AUPR	BCM	CCEM	Rank-sum	Rank
Team036	0.458	0.479	0.509	13	1
Team221	0.454	0.459	0.492	18	2
Team114	0.464	0.443	0.483	20	3
Team063	0.489	0.427	0.489	21	4
Team161	0.496	0.431	0.481	23	5
you	0.421	0.452	0.510	28	6
Team273	0.462	0.431	0.480	29	7
Team227	0.428	0.474	0.480	35	8
Team080	0.447	0.423	0.482	41	9
Team187	0.461	0.440	0.459	43	10
Team245	0.480	0.403	0.477	43	10
Team122	0.481	0.413	0.468	45	12
Team290	0.458	0.408	0.476	45	12
Team132	0.448	0.392	0.487	48	14
Team297	0.496	0.378	0.476	49	15

Plots that present the results of AUPR, BCM and CCEM corresponding to the performance of ***geNetClassifier*** using the option of "**all assigned**".

The RESULTS TABLE placed after these plots presents the values of these parameters and the rank for the top-15 methods.

AUPR

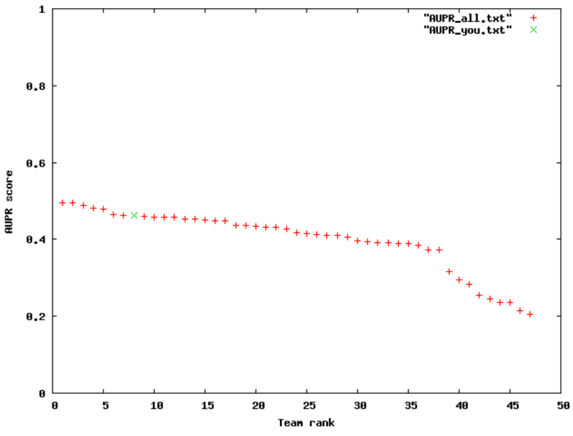

BCM

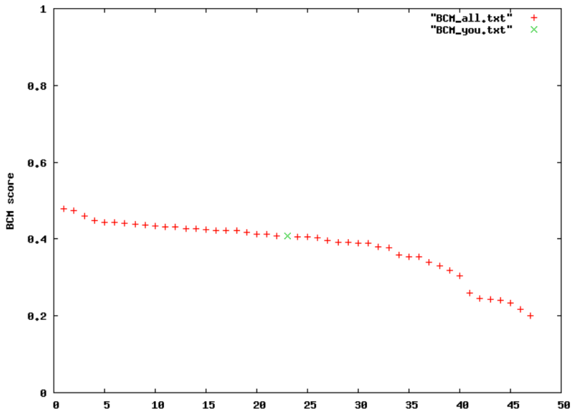

CCEM

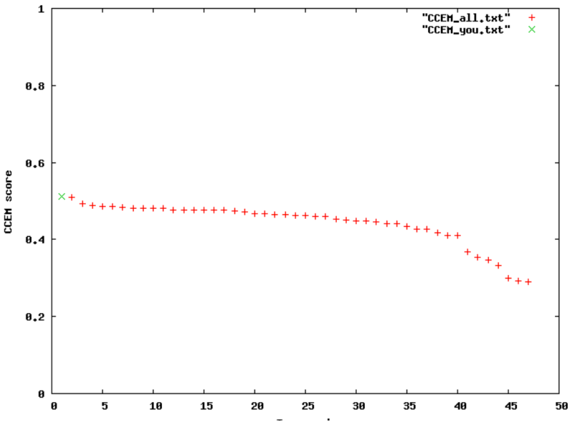

RESULTS TABLE

Team	AUPR	BCM	CCEM	Rank-sum	Rank
Team036	0.458	0.479	0.509	14	1
Team221	0.454	0.459	0.492	19	2
Team114	0.464	0.443	0.483	19	2
Team063	0.489	0.427	0.489	20	4
Team161	0.496	0.431	0.481	22	5
Team273	0.462	0.431	0.480	28	6
you	0.462	0.408	0.511	32	7
Team227	0.428	0.474	0.480	36	8
Team080	0.447	0.423	0.482	41	9
Team187	0.461	0.440	0.459	43	10
Team245	0.480	0.403	0.477	43	10
Team122	0.481	0.413	0.468	44	12
Team290	0.458	0.408	0.476	45	13
Team297	0.496	0.378	0.476	49	14
Team132	0.448	0.392	0.487	49	14
