# Supplementary material for: Analyse multiple disease subtypes and build associated gene networks using genome-wide expression profiles
Source: BMC Genomics. 2015 May 26;16(Suppl 5):S3. doi: 10.1186/1471-2164-16-S5-S3 (PMC4460584; doi:10.1186/1471-2164-16-S5-S3)
Supplement: Additional file 4 — File S4. geNetClassifier vignette including a tutorial with executable examples and description of all the methods. This vignette is available in Bioconductor: http://www.bioconductor.org/packages/release/bioc/vignettes/geNetClassifier/inst/doc/geNetClassifier-vignette.pdf [file 1471-2164-16-S5-S3-S4.pdf]

geNetClassifier
classify multiple diseases and build associated gene networks using gene expression profiles

Sara Aibar, Celia Fontanillo, Conrad Droste, and Javier De Las Rivas

Bioinformatics and Functional Genomics Group
Centro de Investigacion del Cancer (CiC-IBMCC, CSIC/USAL)
Salamanca - Spain

October 24, 2014

Version: 1.6

Contents

1 Introduction to *geNetClassifier* **2**

2 Install the package and example data **4**

3 Main function of the package: *geNetClassifier()* **5**
- 3.1 Loading the package and data 6
- 3.2 Run *geNetClassifier()* 7
- 3.3 Overview of the data returned by *geNetClassifier()* 9
- 3.4 Return I: Genes ranking 10
  - 3.4.1 Significant genes 14
- 3.5 Return II: Classifier 15
  - 3.5.1 Gene selection procedure 16
  - 3.5.2 Estimation of performance and generalization error procedure . . 18
- 3.6 Return III: Gene networks 21

4 External validation: query with new samples of known class **24**
- 4.1 Assignment conditions 26

5 Sample classification: query with new samples of unknown class **28**

6 Functions to plot the results **30**
- 6.1 Plot Ranked Significant Genes: *plot(...@genesRaking)* 30
- 6.2 Plot Gene Expression Profiles: *plotExpressionProfiles()* 31
- 6.3 Plot Genes Discriminant Power: *plotDiscriminantPower()* 33
- 6.4 Plot Gene Networks: *plotNetwork()* 35

1 Introduction to *geNetClassifier*

geNetClassifier is an algorithm designed to build transparent classifiers and the associated gene networks based on genome-wide expression data.

geNetClassifier() is also the name of the main function in the package. This function takes as input the *expressionSet* or expression matrix of the studied samples and the classes the samples belong to (i.e. the diseases or disease subtypes). Once the data are analyzed, geNetClassifier() provides: **(i)** ranked gene sets (or gene signatures) that identify each class; **(ii)** a multiple-class classifier; and **(iii)** gene networks associated to each class.

- Gene ranking: The genes, probesets, or any other variables that are input in the *expressionSet* are considered *features* for the classification. These features are analyzed by *geNetClassifier*, and ranked according to the class they best identify, in order to select the optimum set for training the classifier. This ranking is returned by *geNetClassifier()* as well as the parameters calculated for gene selection.
- Classifier: *geNetClassifier()* also returns a multi-class SVM-based classifier, which can be queried later on; the genes (features) chosen for classification; their discriminant power (a parameter that measures the importance that the classifier internally gives to each gene); and, optionally, the classifier's generalization error and statistics about the selected genes.
- Network: The mutual-information (interactions) and the co-expression (correlations) between the genes are also calculated and analyzed by the algorithm. These allow to estimate the degree of association between the variables and they are used to generate a gene network for each class. These networks can be plotted, providing a integrated overview of the genes that characterized each disease (i.e. each class).

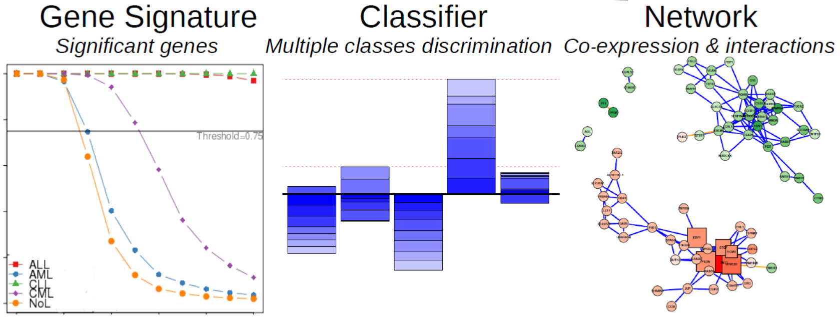

Figure 1. Taking an *expressionSet* as input, *geNetClassifier()* returns a gene signature for each class, a classifier to discriminate the classes, and gene networks associated to each class. The package also includes several analytic and visualizing tools to explore these results.

Examples of use

The algorithm shows a robust performance applied to patient-based gene expression datasets that study disease subtypes or disease classes. In this vignette, we show its performance for a leukemia dataset that includes 60 microarray samples from bone marrow of patients with four major leukemia subtypes (ALL, AML, CLL, CML) and no-leukemia controls (NoL). The results outperform a previously published classification analysis of these data [1].

The method is designed to be applied to the analysis and classification of different disease subtypes. Therefore, in the R package and this vignette, all the explanations and examples are disease-oriented. However, *geNetClassifier* can be applied to the classification of any other type of biological states, pathological or not.

Methods

The algorithm *geNetClassifier()* integrates several existing machine learning and statistical methods. The *feature ranking* is achieved based on a Parametric Empirical Bayes method (PEB). Double-nested internal cross-validation (CV) [2] is used for the *feature selection* process and to estimate the *generalization error* of the classifier. The machine learning method implemented in the classifier is a multi-class Support Vector Machine (SVM) [3]. The gene *networks* are built calculating the relations derived from gene to gene co-expression analysis (by default, *Pearson correlation*) and the interactions derived from gene mutual information analysis (using *minet* package) [4]. More details about these methods are available in the appropriate sections.

Queries

geNetClassifier includes a *query* function that allows either validation of the classifiers using external independent samples of known class (section 4) or classification of new samples whose class is unknown (section 5). This function facilitates the application of the classification algorithm as a predictor for new samples, and it is designed to resemble expert behavior by allowing *NotAssigned* (NA) instances when it is not sure about the class labelling. In order to assign a sample to a class, the algorithm requires a minimum certainty (i.e. probability), leaving it unassigned in case it does not achieve a clear call to a single class. These probability thresholds can be tuned to achieve a more or less stringent assignment. By following this procedure, the algorithm emulates human experts in the decision-making.

2 Install the package and example data

To install *geNetClassifier* from *Bioconductor*:

```
> source("http://bioconductor.org/biocLite.R")
> biocLite("geNetClassifier")
```

To follow the examples presented in this *Vignette*, we also need to install a sample dataset called *leukemiasEset*:

```
> biocLite("leukemiasEset")
```

This dataset contains an *expresssionSet* built with 60 gene expression microarrays (HG-U133 plus 2.0 from *Affymetrix*) hybridized with mRNA extracted from bone marrow biopsies of patients of the 4 major types of leukemia (ALL, AML, CLL and CML) and from non-leukemia controls (NoL). These data was produced by the Microarray Innovations in LEukemia (MILE) research project [1] and are available at GEO, under accession number GSE13159. The selected samples are labeled keeping their source GEO IDs.

To have an overview of this *ExpressionSet* and its available info:

```
> library(leukemiasEset)
> data(leukemiasEset)
> leukemiasEset

ExpressionSet (storageMode: lockedEnvironment)
assayData: 20172 features, 60 samples
  element names: exprs, se.exprs
protocolData
  sampleNames: GSM330151.CEL GSM330153.CEL ... GSM331677.CEL (60 total)
  varLabels: ScanDate
  varMetadata: labelDescription
phenoData
  sampleNames: GSM330151.CEL GSM330153.CEL ... GSM331677.CEL (60 total)
  varLabels: Project Tissue ... Subtype (5 total)
  varMetadata: labelDescription
featureData: none
experimentData: use 'experimentData(object)'
Annotation: genemapperhgu133plus2

> summary(leukemiasEset$LeukemiaType)

ALL AML CLL CML NoL
 12  12  12  12  12

> pData(leukemiasEset)
```

For further information/help about this *ExpressionSet*:

```
> ?leukemiasEset
```

CEL files were preprocessed using an alternative Chip Description File (CDF), which allows mapping the expression directly to genes (Ensembl IDs ENSG) instead of *Affymetrix* probesets. This alternative CDF, with redefines gene-based annotation files for the *Affymetrix* expression microarrays, can be found in *GATExplorer* (a bioinformatic web platform that integrates a gene loci browser with nucleotide level re-mapping of the oligo *probes* from *Affymetrix* expression microarrays to genes: mRNAs and ncRNAs)[5].

To translate these Ensembl gene IDs into Gene Symbols for easier reading, the optional argument *geneLabels* from *geNetClassifier* can be used. This option allows to extend the annotation and labelling of the genes by providing a table that contains the gene symbol and other characteristics of the genes in the *expresssionSet*. This option can be used with any annotation (i.e. Bioconductor's *org.Hs.eg.db* package) as long as it is provided in the correct format. However, for increased consistency between versions, when using *GATExplorer* CDF, we recomend to also use *GATExplorer* annotation files. Annotation files with the Gene Symbol corresponding to each Ensembl gene ID can be found at: `http://bioinfow.dep.usal.es/xgate/mapping/mapping.php?content=annotationfiles`. The one used in this example is the *Human Genes* R annotation file. A subset of this file was saved into the object *geneSymbols* for easier use in the examples:

```
> data(geneSymbols)
> head(geneSymbols)
```

This annotation file provides further information which can be used to filter the genes. i.e. To consider only protein-coding genes for the construction of *geNetClassifier*, use the following filter:

```
> load("genes-human-annotation.R")
> leukEset_protCoding <- leukemiasEset[featureNames(leukemiasEset)
+ %in% rownames(genes.human.Annotation[genes.human.Annotation$biotype
+ %in% "protein_coding",]),]
> dim(leukemiasEset)
> dim(leukEset_protCoding)
```

Please note that *geNetClassifier* is designed to work with genes. In case the expression data is not summarized into genes (i.e. it uses the default *probesets*) *geNetClassifier* can still be used but those probesets/features will still be called *genes*.

3 Main function of the package: *geNetClassifier()*

geNetClassifier() is the main function of the package. It builds the classifier and the gene network associated to each class, and also returns the genes ranking and further information about the selected genes.

The workflow internally followed by *geNetClassifier()* includes the following steps:

1.- Filtering data and calculating the genes ranking.
2.- Calculating correlations between genes.
3.- Calculating interactions between genes.
Optional - Filter of redundant genes from the ranking (see arguments *removeCorrelations* and *removeInteractions*).

4.- Construction of the classifier: Selects of a subset of genes to train the classifier through 8-fold cross-validation. The selected genes are used to train the classifier with the complete set of samples.
5.- Estimation of performance: calculates the *generalization error* of the classifier and the statistics about the genes adding an 5-fold *cross-validation* around the construction of the classifier (*nested cross-validation*).
6.- Construction of the gene networks: a gene network is built for each one of the classes using the pairwise gene-to-gene correlations and interactions.
7.- Writing and saving the results including a series of plots for visualization.

The following sections show: how to load the package and the data (sec. 3.1); how to run the algorithm (sec. 3.2); an overview of the results and returned data (sec. 3.3): the genes ranking (sec. 3.4), the classifier (sec. 3.5) and the gene networks (sec. 3.6).

3.1 Loading the package and data

In order to have *geNetClassifier* functions available, the first step is to load the package:

```
> library(geNetClassifier)
```

To list all available tutorials for this package, or to open this *Vignette* you can use:

```
> # List available vignettes for package geNetClassifier:
> vignette(package="geNetClassifier")
> # Open vignette named "geNetClassifier-vignette":
> vignette("geNetClassifier-vignette")
```

To list all the available functions and objects included in *geNetClassifier* use the function *objects()*. Typing its name with a question mark (?) before any function, will show its help file. Through this tutorial, we will see how to use the main ones:

```
> objects("package:geNetClassifier")
```

```
 [1] "calculateGenesRanking"        "externalValidation.probMatrix"
 [3] "externalValidation.stats"     "gClasses"
 [5] "genesDetails"                 "geNetClassifier"
 [7] "getEdges"                     "getNodes"
 [9] "getNumEdges"                  "getNumNodes"
[11] "getRanking"                   "getSubNetwork"
[13] "getTopRanking"                "initialize"
[15] "network2txt"                  "numGenes"
[17] "numSignificantGenes"          "overview"
[19] "plotAssignments"              "plotDiscriminantPower"
[21] "plotExpressionProfiles"       "plot.GenesNetwork"
[23] "plot.GenesRanking"            "plot.GeNetClassifierReturn"
[25] "plotNetwork"                  "queryGeNetClassifier"
[27] "querySummary"                 "setProperties"
[29] "show"
```

```
> ?geNetClassifier
```

After the package is loaded, you can proceed to analyze your data. In this vignette we use *leukemiasEset*: 60 microarrays from bone marrow from patients of the 4 major types of leukemia (ALL, AML, CLL, CML) and from healthy non-leukemia controls (NoL).(For installation and further information regarding *leukemiasEset* data package see Section 2).

```
> library(leukemiasEset)
> data(leukemiasEset)
```

In *leukemiasEset* there are 60 samples: 12 of each class (ALL, AML, CLL, CML and NoL). We will select 10 samples from each class to execute *geNetClassifier()*, and leave 2 for external validation of the resulting classifier. In this way, it makes a total of 50 samples for the *training* and 10 samples for the *validation*.

```
> trainSamples <- c(1:10, 13:22, 25:34, 37:46, 49:58)
> summary(leukemiasEset$LeukemiaType[trainSamples])

ALL AML CLL CML NoL
 10  10  10  10  10
```

3.2 Run *geNetClassifier()*

The essential input elements that *geNetClassifier* needs are:

1.- An *expressionSet*: R object defined in Bioconductor that contains a genome-wide expression matrix with data for multiple samples; see *?ExpressionSet*. Note that since the ranking is built though package *EBarrays*, the data in the expression set should be normalized intensity values (positive and on raw scale, not on a logarithmic scale).

2.- The *sampleLabels*: a vector with the class name of each sample or the *ExpressionSet phenoData* object containing this information. Note that to run *geNetClassifier* it is highly recommended to have the **same number of samples in each class**. A balanced number of samples allows an even exploration of each class and provides better classification.

The algorithm input also includes many other arguments that allow to personalize the execution or modify some of the parameters internally used. All of them have a default value and there is no need to modify them. In the following step we will see examples on how to use the main ones. Information about them can be found using the help options (i.e. *?geNetClassifier*). This is the full list of arguments with their default values:

```
geNetClassifier(eset, sampleLabels, plotsName=NULL, buildClassifier=TRUE,
estimateGError=FALSE, calculateNetwork=TRUE, labelsOrder=NULL,
geneLabels=NULL, numGenesNetworkPlot=100, minGenesTrain=1,
maxGenesTrain=100, continueZeroError=FALSE, numIters=6, lpThreshold=0.95,
numDecimals=3, removeCorrelations=FALSE, correlationsThreshold=0.8,
correlationMethod="pearson", removeInteractions=FALSE, interactionsThreshold=0.5,
skipInteractions=FALSE, minProbAssignCoeff=1, minDiffAssignCoeff=0.8,
IQRfilterPercentage=0, precalcGenesNetwork=NULL, precalcGenesRanking=NULL,
returnAllGenesRanking=TRUE, verbose=TRUE)
```

The execution time will depend on the computer and the size of the dataset. To avoid waiting now for the construction of a new classifier to continue this tutorial, a pre-executed example is included in the package:

```
> data(leukemiasClassifier)
```

This classifier was built running the following code:

```
> leukemiasClassifier <- geNetClassifier(leukEset_protCoding[,trainSamples],
+ sampleLabels="LeukemiaType", plotsName="leukemiasClassifier",
+ estimateGError=TRUE, geneLabels=geneSymbols)
```

These are some examples of standard use:

- The fastest execution would be training the classifier exploring a reduced number of genes (by default *maxGenesTrain=100*). In order ot skip calculating the network within the genes, set *calculateNetwork=FALSE*. However, since the correlations are relatively fast to calculate, we recommend keeping *calculateNetwork=TRUE*, and set *skipInteractions=TRUE* instead.

  ```
  > leukemiasClassifier <- geNetClassifier(eset=leukemiasEset[,trainSamples],
  + sampleLabels="LeukemiaType", plotsName="leukemiasClassifier",
  + skipInteractions=TRUE, maxGenesTrain=20, geneLabels=geneSymbols)
  ```

- The default execution (*buildClassifier=TRUE*, *calculateNetwork=TRUE*) only requires the *expressionSet* and the *sampleLabels*. Providing *plotsName* is also recommended in order to produce the plots:

  ```
  > leukemiasClassifier <- geNetClassifier(eset=leukemiasEset[,trainSamples],
  + sampleLabels="LeukemiaType", plotsName="leukemiasClassifier")
  ```

- In order to also estimate the classifier's performance, set *estimateGError=TRUE*. This option will take longer to execute

  ```
  > leukemiasClassifier <- geNetClassifier(eset=leukemiasEset[,trainSamples],
  + sampleLabels="LeukemiaType", plotsName="leukemiasClassifier",
  + estimateGError=TRUE)
  ```

Some of the parameters allow to provide extra information for an easier reading of the results:
- *labelsOrder* allows to show and plot the classes in a specific order (i.e. *labelsOrder=c('ALL', 'CLL', 'AML', 'CML', 'NoL')*)
- *geneLabels* can be used to add a label to the genes to show in the outputs instead of the *featureNames* from the *ExpressionSet*.
In the example, the genes were labeled with the gene symbols provided by *GATExplorer* gene-based probe mapping (*geneLabels=geneSymbols*), as it was indicated in section 3.1.

After running *geNetClassifier()*, we recommend to save the output:

```
> getwd()
> save(leukemiasClassifier, file="leukemiasClassifier.RData")
```

3.3 Overview of the data returned by *geNetClassifier()*

The main results that *leukemiasClassifier()* provides are: the **genes ranking** (sec. 3.4), the **classifier** (sec.3.5) and the **gene networks** (sec. 3.6). All this information is returned by *geNetClassifier()* in an object of class *GeNetClassifierReturn*. This objec tontains several slots which can be seen with the function names():

```
> names(leukemiasClassifier)

[1] "call"                "classifier"
[3] "classificationGenes" "generalizationError"
[5] "genesRanking"        "genesRankingType"
[7] "genesNetwork"        "genesNetworkType"
```

The slot **@call** contains the R sentence that was used to execute *geNetClassifier()*. It is the only slot that will always be returned by *geNetClassifier()*, the presence and contents of the other components returned by the algorithm will depend on the arguments used to run it.

```
> leukemiasClassifier@call

geNetClassifier(eset = leukEset_protCoding[, trainSamples], sampleLabels = "LeukemiaT
    plotsName = "leukemiasClassifier", buildClassifier = TRUE,
    estimateGError = TRUE, calculateNetwork = TRUE, geneLabels = geneSymbols)
```

All the outputs and returned components are explained in detail in the following sections:

- @**genesRanking** in section 3.4
- @**classifier** and @**classificationGenes** in section 3.5
- @**generalizationError** in section 3.5.2
- @**genesNetwork** in section 3.6
- The **plots** are explained in section 6

A general view of the output can be seen by just typing the assigned name:

```
> leukemiasClassifier

R object summary:
Classifier trained with 50 samples.
Total number of genes included in the classifier: 26.
Number of genes per class:
ALL AML CLL CML NoL
  9   5   1   5   6
For classificationGenes details: genesDetails(EXAMPLE@classificationGenes)

Generalization error and gene stats calculated through 5-fold cross-validation:
[1] "accuracy"                  "sensitivitySpecificity"
[3] "confMatrix"                "probMatrix"
[5] "querySummary"              "classificationGenes.stats"
```

```
[7] "classificationGenes.num"

The ranking of all genes contains (genes per class):
 ALL  AML  CLL  CML  NoL
2342 3023 2824 2539 3049

The networks calculated for the topGenes genes of each class contain:
                     ALL AML   CLL  CML  NoL
Number of genes     1027 400  1916  949  400
Number of relations 1942 296 18506 6540 1993

Available slots in this R object:
[1] "call"                "classifier"          "classificationGenes"
[4] "generalizationError" "genesRanking"        "genesRankingType"
[7] "genesNetwork"        "genesNetworkType"
To see an overview of all available slots type "overview(EXAMPLE)"
```

3.4 Return I: Genes ranking

The first step of *geNetClassifier* algorithm is to determine a ranking of genes for each class based in the analysis of the expression signal. To create this ranking, it uses the function *emfit*, a Parametric Empirical Bayes method [6], included in package *EBarrays* [7]. This method implements an expectation-maximization (EM) algorithm for gene expression mixture models, which compares the patterns of differential expression across multiple conditions and provides a *posterior probability*.

The posterior probability is calculated for each gene-class pair, and represents how much each gene differentiates a class from the other classes; being 1 the best value, and 0 the worst. In this way, the posterior probability allows to find the genes that show significant differential expression when comparing the samples of one class *versus* all the other samples (One-versus-Rest comparison).

A first version of the ranking is built by ordering the genes decreasingly by their posterior probability for each class. To resolve the ties, *geNetClassifier* uses the expression difference between the mean for each gene in the given class and the mean in the closest class. In addition, the genes with a posterior probability greater or equal to 0.95 for the 'no difference' -the genes that do not show any difference between classes- are filtered out before proceeding into further steps.

The final version of the ranking is built assigning each gene to the class in which it has the best ranking. In this way the separation between classes is optimized, and the method will choose first the genes that best differentiate any of the classes. As a result of this process, even if a gene is found associated to several classes during the expression analysis, **each gene can only be on the ranking of one class**.

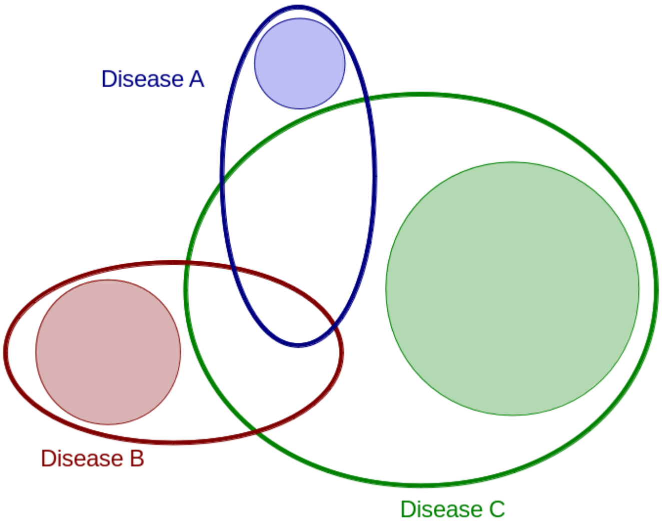

Figure 2. Scheme representing the overlap between the sets of genes that each disease may affect. *geNetClassifier* explores all the genes that affect each disease (**ovals**) and selects as significant, the genes that are unique (differentially expressed) to each disease (**coloured circles**).

The genes ranking obtained for each class is used for the gene selection in the classification procedure and it is also provided as an output of *geNetClassifier()* in the slot: ...@genesRanking.

```
> leukemiasClassifier@genesRanking

Top ranked genes for the classes:  ALL AML CLL CML NoL
      ALL       AML      CLL        CML       NoL
 [1,] "VPREB1"  "HOXA9"  "TYMS"     "GJB6"    "FGF13"
 [2,] "ZNF423"  "MEIS1"  "FCER2"    "PRG3"    "NMU"
 [3,] "DNTT"    "CD24L4" "NUCB2"    "LY86"    "SMPDL3A"
 [4,] "EBF1"    "ANGPT1" "RRAS2"    "ABP1"    "KLRB1"
 [5,] "PXDN"    "CCNA1"  "PNOC"     "TRIM22"  "RNF182"
 [6,] "S100A16" "ZNF521" "C6orf105" "NLRC3"   "RFESD"
 [7,] "CSRP2"   "HOXA5"  "RRM2"     "LPXN"    "SLC25A21"
 [8,] "SOCS2"   "DEPDC6" "KIAA0101" "GBP3"    "CD160"
 [9,] "CTGF"    "NKX2-3" "UHRF1"    "TNS3"    "CLIC2"
[10,] "COL5A1"  "NPTX2"  "ABCA6"    "ZC3H12D" "TMEM56"
...

Number of ranked significant genes (posterior probability over threshold):
         ALL AML CLL CML NoL
         1027 273 1916 949 191
To see the whole ranking (3049 rows) use: getRanking(...)
Details of the top X ranked genes of each class: genesDetails(..., nGenes=X)
```

This ranking an object of class *GenesRanking*. This class provides some utility functions which will help working with the information contained in the object. The total number

of genes in the ranking for each class can be queried using the function *numGenes()*. These numbers include all the genes that have some ability to distinguish between classes, although only the top ones are really significant.

```
> numGenes(leukemiasClassifier@genesRanking)

 ALL  AML  CLL  CML  NoL
2342 3023 2824 2539 3049
```

With *getTopRanking()* a subset of the ranking containing only the given number of top genes can be obtained. Since the returned object is also a *GenesRanking* object, no information is lost and other functions (i.e. *genesDetails()*) can be used afterwards.

```
> subRanking <- getTopRanking(leukemiasClassifier@genesRanking, 10)
```

In order to retrieve the whole ranking in the form of a matrix (i.e. to print the full version or get a subset of it), the function *getRanking()* can be used. This function provides the option to show the ranking with the gene IDs or the gene Labels.

```
> getRanking(subRanking)

$geneLabels
      ALL       AML      CLL        CML       NoL
 [1,] "VPREB1"  "HOXA9"  "TYMS"     "GJB6"    "FGF13"
 [2,] "ZNF423"  "MEIS1"  "FCER2"    "PRG3"    "NMU"
 [3,] "DNTT"    "CD24L4" "NUCB2"    "LY86"    "SMPDL3A"
 [4,] "EBF1"    "ANGPT1" "RRAS2"    "ABP1"    "KLRB1"
 [5,] "PXDN"    "CCNA1"  "PNOC"     "TRIM22"  "RNF182"
 [6,] "S100A16" "ZNF521" "C6orf105" "NLRC3"   "RFESD"
 [7,] "CSRP2"   "HOXA5"  "RRM2"     "LPXN"    "SLC25A21"
 [8,] "SOCS2"   "DEPDC6" "KIAA0101" "GBP3"    "CD160"
 [9,] "CTGF"    "NKX2-3" "UHRF1"    "TNS3"    "CLIC2"
[10,] "COL5A1"  "NPTX2"  "ABCA6"    "ZC3H12D" "TMEM56"

> getRanking(subRanking, showGeneID=TRUE)$geneID[,1:4]

      ALL               AML               CLL               CML
 [1,] "ENSG00000169575" "ENSG00000078399" "ENSG00000176890" "ENSG00000121742"
 [2,] "ENSG00000102935" "ENSG00000143995" "ENSG00000104921" "ENSG00000156575"
 [3,] "ENSG00000107447" "ENSG00000185275" "ENSG00000070081" "ENSG00000112799"
 [4,] "ENSG00000164330" "ENSG00000154188" "ENSG00000133818" "ENSG00000002726"
 [5,] "ENSG00000130508" "ENSG00000133101" "ENSG00000168081" "ENSG00000132274"
 [6,] "ENSG00000188643" "ENSG00000198795" "ENSG00000111863" "ENSG00000167984"
 [7,] "ENSG00000175183" "ENSG00000106004" "ENSG00000171848" "ENSG00000110031"
 [8,] "ENSG00000120833" "ENSG00000155792" "ENSG00000166803" "ENSG00000117226"
 [9,] "ENSG00000118523" "ENSG00000119919" "ENSG00000034063" "ENSG00000136205"
[10,] "ENSG00000130635" "ENSG00000106236" "ENSG00000154262" "ENSG00000178199"
```

The function *genesDetails()* allows to show all the available info of the genes in the ranking.

```
> genesDetails(subRanking)$AML

                GeneName ranking class postProb exprsMeanDiff exprsUpDw
ENSG00000078399    HOXA9       1   AML        1        4.4362        UP
ENSG00000143995    MEIS1       2   AML        1        3.2785        UP
ENSG00000185275   CD24L4       3   AML        1       -4.4926      DOWN
ENSG00000154188   ANGPT1       4   AML        1        2.7427        UP
ENSG00000133101    CCNA1       5   AML        1        2.5558        UP
ENSG00000198795   ZNF521       6   AML        1        2.5697        UP
ENSG00000106004    HOXA5       7   AML        1        3.1729        UP
ENSG00000155792   DEPDC6       8   AML        1        2.4803        UP
ENSG00000119919   NKX2-3       9   AML        1        2.1962        UP
ENSG00000106236    NPTX2      10   AML        1        2.0582        UP
                isRedundant
ENSG00000078399       FALSE
ENSG00000143995        TRUE
ENSG00000185275       FALSE
ENSG00000154188       FALSE
ENSG00000133101       FALSE
ENSG00000198795        TRUE
ENSG00000106004        TRUE
ENSG00000155792        TRUE
ENSG00000119919       FALSE
ENSG00000106236       FALSE
```

NOTE: If the console splits the table into several lines, try:

```
> options(width=200)
```

By default, the *rownames* are the ID included in the *expressionSet*: in our case the ENSEMBL IDs. The *GeneName* column has been added by setting the argument *geneLabels=geneSymbols* (see sec. 3.2).

To see the description of the content of this table write: *?genesDetails*.

More details about *GenesRanking* class is available at: *?GenesRanking*.

3.4.1 Significant genes

The set of genes considered *significant* for each of the classes is determined by a common threshold for the posterior probability (by default *lpThreshold=0.95*). This common threshold provides a way to quantify the size of the *gene signature* assigned to each disease (as always: compared to the other diseases in the study). In this way, the algorithm provides a framework to compare biological states, i.e. the biological or pathological conditions represented in the samples.

plotSignificantGenes() provides a plot of the distribution of the posterior probabilities of the genes within the rankings for each class:

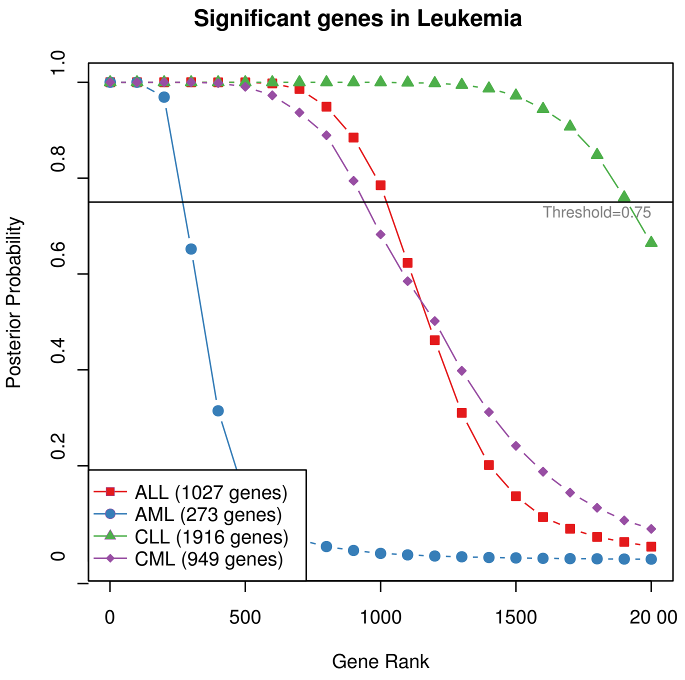

Figure 3. Plot of the posterior probabilities of the genes of 4 leukemia classes, ordering the genes according to their rank.

This example shows the big differences in size of the gene sets assigned to a disease: at lpThreshold 0.95 CLL has been assigned 2028 genes, while AML only 308 genes. The biological interpretation of this observation will depend on the specific study. Larger gene signatures may be an indication of more *systemic* diseases (i.e. a disease affect more genes than another), but it may also be an indication of the relative differences between the diseases in the study (i.e. one of the diseases affects different genes than the others). In any case, the results provided by *geNetClassifier* may help to unravel disease sub-types differences based on the gene signatures.

numSignificantGenes() provides the number of significant genes, the number of genes with posterior probability over the threshold:

```
> numSignificantGenes(leukemiasClassifier@genesRanking)

 ALL  AML  CLL  CML  NoL
1027  273 1916  949  191
```

The plot of the posterior probability (*plotSignificantGenes()*) is the default plot for objects of class *GenesRanking*. (More details in section 6.1).

```
> plot(leukemiasClassifier@genesRanking)
```

In both functions, the threshold can be modified through *lpThreshold*:

```
> plot(leukemiasClassifier@genesRanking,
+ numGenesPlot=3000, lpThreshold=0.80)
```

3.5 Return II: Classifier

The information regarding the classifier is saved into the slots: @classifier, @classifcationGenes and @generalizationError.

The *@**classifier*** slot contains the SVM classifier that can later be used to make queries. The SVM method included in the algorithm is a linear kernel implementation from R package *e1071*. This implementation allows multi-class classification by using a One-versus-One (OvO) approach, in which all the binary classifications are fitted and the correct class is found based on a voting system.

```
> leukemiasClassifier@classifier

$SVMclassifier

Call:
svm.default(x = t(esetFilteredDataFrame[buildGenesVector, trainSamples]),
    y = sampleLabels[trainSamples], kernel = "linear", probability = T,
    C = 1)


Parameters:
   SVM-Type:  C-classification
 SVM-Kernel:  linear
       cost:  1
      gamma:  0.03846154

Number of Support Vectors:  29
```

*@**classificationGenes*** contains the final genes selected to build the classifier. Since *@classificationGenes* is an object of class *GenesRanking*, functions such as *numGenes()* or *genesDetails()* can be used to explore it.

```
> leukemiasClassifier@classificationGenes

Top ranked genes for the classes:  ALL AML CLL CML NoL
      ALL      AML      CLL    CML      NoL
 [1,] "VPREB1" "HOXA9"  "TYMS" "GJB6"   "FGF13"
 [2,] "ZNF423" "MEIS1"  NA     "PRG3"   "NMU"
 [3,] "DNTT"   "CD24L4" NA     "LY86"   "SMPDL3A"
 [4,] "EBF1"   "ANGPT1" NA     "ABP1"   "KLRB1"
```

```
 [5,] "PXDN"    "CCNA1"  NA     "TRIM22" "RNF182"
 [6,] "S100A16" NA       NA     NA       "RFESD"
 [7,] "CSRP2"   NA       NA     NA       NA
 [8,] "SOCS2"   NA       NA     NA       NA
 [9,] "CTGF"    NA       NA     NA       NA

Details of the top X ranked genes of each class: genesDetails(..., nGenes=X)
```

```
> numGenes(leukemiasClassifier@classificationGenes)
```

```
ALL AML CLL CML NoL
  9   5   1   5   6
```

```
> genesDetails(leukemiasClassifier@classificationGenes)$ALL
```

```
                GeneName ranking gERankMean class postProb exprsMeanDiff
ENSG00000169575   VPREB1       1        1.0   ALL        1        6.3307
ENSG00000102935   ZNF423       2        3.0   ALL        1        5.0980
ENSG00000107447     DNTT       3        2.8   ALL        1        6.8948
ENSG00000164330     EBF1       4        3.8   ALL        1        5.4171
ENSG00000130508     PXDN       5        5.2   ALL        1        5.0387
ENSG00000188643  S100A16       6        5.4   ALL        1        4.3434
ENSG00000175183    CSRP2       7        7.8   ALL        1        4.0479
ENSG00000120833    SOCS2       8       10.8   ALL        1        4.5383
ENSG00000118523     CTGF       9       14.8   ALL        1        3.6167
                exprsUpDw discriminantPower discrPwClass isRedundant
ENSG00000169575        UP          9.416945          ALL       FALSE
ENSG00000102935        UP         13.240579          ALL        TRUE
ENSG00000107447        UP          8.978735          ALL        TRUE
ENSG00000164330        UP         10.515557          ALL        TRUE
ENSG00000130508        UP          8.657167          ALL        TRUE
ENSG00000188643        UP         12.385161          ALL        TRUE
ENSG00000175183        UP          8.782649          ALL        TRUE
ENSG00000120833        UP          8.697958          ALL       FALSE
ENSG00000118523        UP          5.551344          ALL       FALSE
```

Note that besides the common information about the genes provided by the genes ranking (sec. 3.4), the classification genes also have information about the **discriminant power** of the genes (sec. 6.3).

For details on the *gene selection procedure* (sec. 3.5.1) and the *estimation of performance and generalization error procedure* (slot *@**generalization***) (sec. 3.5.2), see the next two sections.

3.5.1 Gene selection procedure

The optimum number of genes to train the classifier is selected by evaluating the classifiers trained with increasing number of genes. This is done using several iterations of 8-fold cross-validation. Each cross-validation iteration starts with the first ranked gene of each class: it trains an internal classifier with these genes, and evaluates its performance.

One more gene is added in each step to those classes for which a 'perfect prediction' is not achieved (i.e. not all samples correctly identified). The genes are taken in order from the *genes ranking* of each class until any of the classes reaches gets to the maximum number of genes (*maxGenesTrain=100*) or until zero error is reached (*continueZeroError=FALSE*). The error for each of the classifiers and the number of genes used to construct them are saved. Once the cross-validation loop is finished, it saves the minimum number of genes per class which produced the classifier with minimum error.

To achieve the best stability in the number of selected genes, the cross-validation is not run just once, but it is repeated several times with new samplings. This process is repeated as many times as indicated by the optional parameter *numIters* (6 by default). In each of these iterations, the minor number of genes that provided the smallest error is selected.

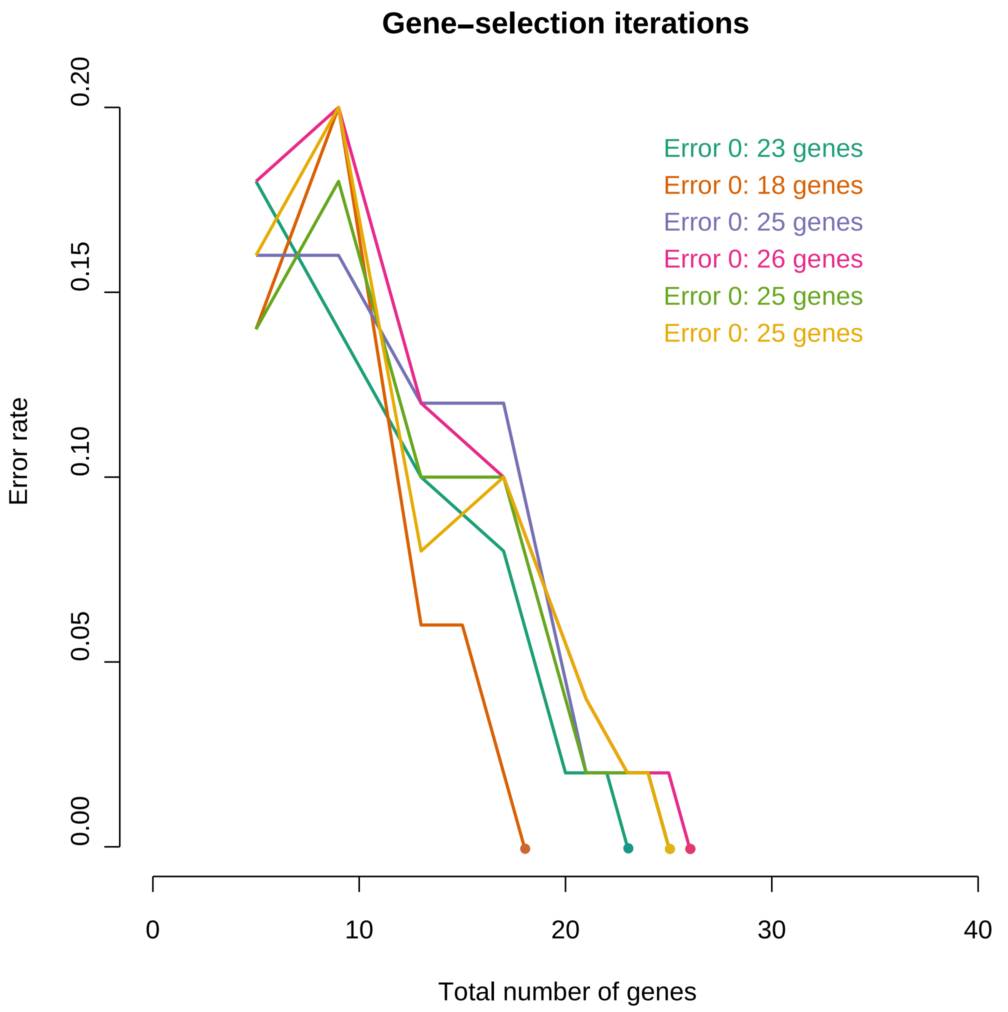

Figure 4. Plot of the gene-selection iterations. Each line represents an iteration and the error rates observed for each number of genes (starting at 5, one per class). The algorithm runs until exploring a maximum number of genes in any class (*maxGeneTrain=100*) or until zero error is reached (*continueZeroError=FALSE*). In each iteration the minimum number of genes with minimum error is selected.

The final selection is done based on the genes selected in each of the iterations. For each class, the top ranked genes are selected by taking the highest number of genes –excluding outliers– selected in the cross-validaton iterations. This allows to identify a stable number of genes, while accounting for the diffences in sampling.

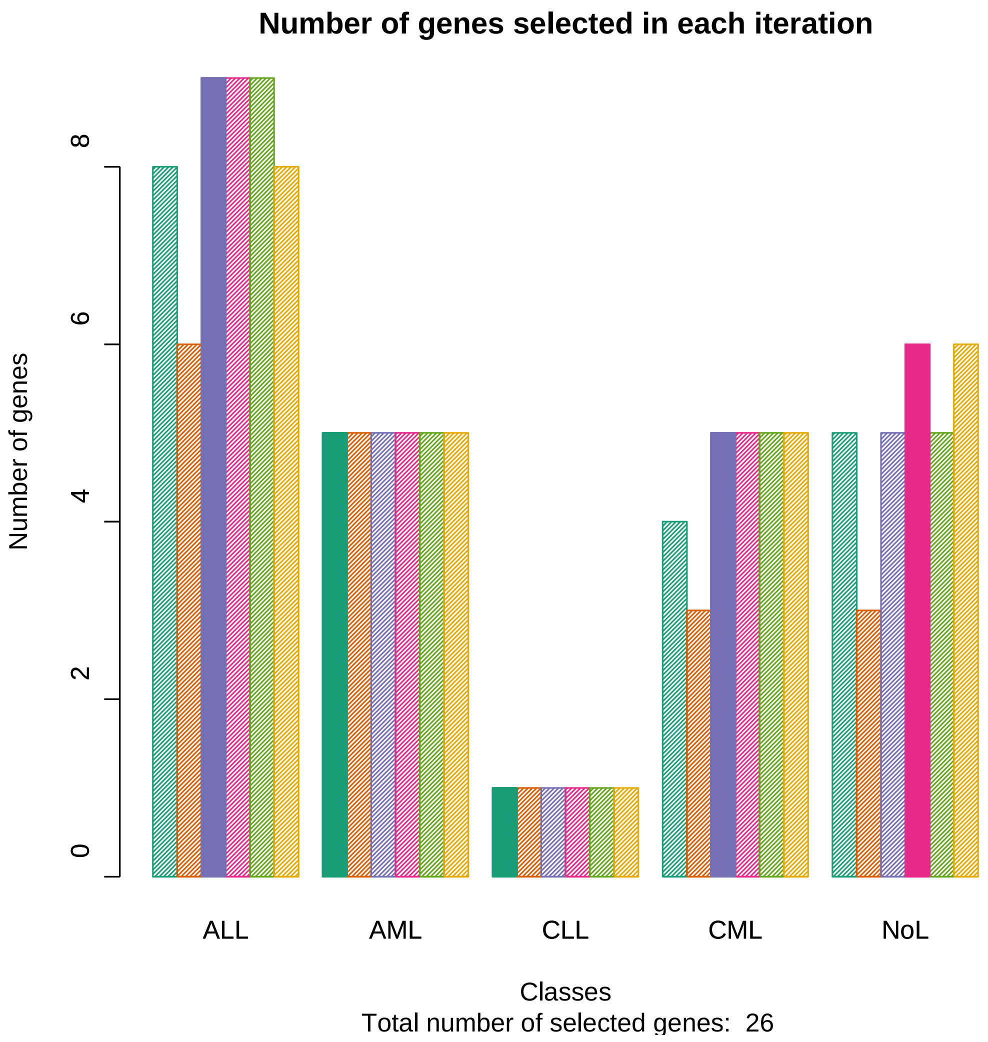

Figure 5. Plot of the number of genes selected in each iteration. The bars represent the number of genes with minimum error rates in each iteration. Each color represents an iteration. The filled bar is the final number of genes of each class selected to train the classifier.

Figures 4 and 5 show the gene selection for the leukemia's example.

3.5.2 Estimation of performance and generalization error procedure

The estimation of the *generalization error* (GE) of the classification algorithm is an option that can be included using the parameter *estimateGError=TRUE*. When this option is chosen, an independent validation is simulated by adding a second loop of cross-validation (CV) around the construction of the classifier. In each iteration of this loop, a few sam-

ples are left out of the *training* and used as *test* samples. This step allows to estimate and provide statistics and metrics regarding the quality of the classifier and the genes selected for classification. The parameters measured for the classifier are the following:

- **Sensitivity**: Proportion of samples from a given class which were correctly identified. In statistical terms it is the rate of true positives (TP). *Sensitivity* relates to the ability of the test to identify positive results.

$$Sensitivity = \frac{TP}{TP + FN} = TruePositiveRate$$

- **Specificity**: Proportion of samples assigned to a given class which really belonged to the class. In statistical terms it is the rate of true negatives (TN). *Specificity* relates to the ability of the test to identify negative results.

$$Specificity = \frac{TN}{TN + FP} = TrueNegativeRate$$

Note: In order to truly evaluate the classification, both sensitivity and specificity need to be taken into account. For example, 100% sensitivity for AML will be achieved by assigning all AML samples to AML. In the same way, 100% specificity will be achieved by not assigning any sample from other class to AML. Therefore, the classification will only be reliable if both -sensitivity and specificity- are optimized, by identifying all samples from one class while not having samples from another classes miss-classified.

- **Matthews Correlation Coefficient** (MCC): It is a measure which takes into account both true and false positives and negatives. It is generally regarded as a balanced measure of performance. In machine learning it is used as a measure of the quality of binary classifications.

$$MCC = \frac{TP \times TN - FP \times FN}{\sqrt{(TP + FP)(TP + FN)(TN + FP)(TN + FN)}}$$

- **Global Accuracy**: Proportion of true results within the assigned samples.

- **Call Rate per class and Global Call Rate**: Proportion of *assigned* samples within a class or in the whole prediction.

$$CallRate = \frac{Assigned}{Assigned + NotAssigned}$$

The results about the estimation of performance and the generalization error are saved in the slot: *@generalizationError*

```
> leukemiasClassifier@generalizationError

Estimated accuracy, sensitivity and specificity for the classifier:
       Accuracy CallRate
Global      100       90
    Sensitivity Specificity MCC CallRate
ALL         100         100 100       90
```

```
AML         100          100 100        70
CLL         100          100 100       100
CML         100          100 100       100
NoL         100          100 100        90

To see all available statistics type "overview(EXAMPLE@generalizationError)"
```

To see all the available info gathered during estimation of performance use the *overview()* function:

```
> overview(leukemiasClassifier@generalizationError)
```

This object contains all the information regarding estimation of performance in different slots: *@accuracy*, *@sensitivitySpecificity*, *@confMatrix*, *@probMatrix*, *@querySummary*.

The slot *...@confMatrix* contains the confusion matrix. A confusion matrix is a table used to quickly visualize and evaluate the performance of a classification algorithm. The rows represent the real class of the samples, while the columns represent the class to which the samples were assigned. Therefore, the correctly assigned samples are in the diagonal.

```
> leukemiasClassifier@generalizationError@confMatrix

          prediction
testLabels ALL AML CLL CML NoL NotAssigned
       ALL   9   0   0   0   0           1
       AML   0   7   0   0   0           3
       CLL   0   0  10   0   0           0
       CML   0   0   0  10   0           0
       NoL   0   0   0   0   9           1
```

The slot *...@probMatrix* presents the probabilities of assignment to each class that are calculated during the 5-fold cross-validation. This *probability matrix* provides a good estimation of how easy or difficult is to assign each sample to its class. It also provides an indication about the likelihood to confuse one class with others:

```
> leukemiasClassifier@generalizationError@probMatrix

      ALL   AML   CLL   CML   NoL
ALL 0.697 0.060 0.073 0.067 0.102
AML 0.058 0.770 0.083 0.044 0.045
CLL 0.088 0.094 0.673 0.064 0.080
CML 0.055 0.107 0.064 0.633 0.141
NoL 0.073 0.072 0.055 0.145 0.654
```

The slot *...@classificationGenes.stats* includes calculations about the number of times that each gene was selected for classification in the 5-fold cross-validation executions:
- *timesChosen*, number of times that each gene is chosen for classification in the 5 CV.
- *chosenRankMean*, average rank of the gene only within the CV loops in which the gene was chosen for classification.
- *chosenRankSD*, standard deviation of the gene rank only within the CV loops in which the gene was chosen for classification.

- *geRankMean*, average rank of the gene in the 5 CV loops performed during the generalization error estimation.
- *geRankSD*, standard deviation of the rank of the gene in the 5 CV loops performed during the generalization error estimation.

```
> leukemiasClassifier@generalizationError@classificationGenes.stats$CLL

                timesChosen chosenRankMean chosenRankSD gERankMean gERankSD
ENSG00000176890           4           1.25         0.50        1.8     1.30
ENSG00000070081           2           1.50         0.71        2.4     0.89
ENSG00000104921           1           1.00         0.00        2.8     1.48
```

The slot *...@classificationGenes.num* includes calculations about the number of genes selected for each class in the 5 runs of the 5-fold cross-validation applied for the estimation of performance. These numbers allow to explore the number of genes that are used per class. However, the proper calculation of the final *number of genes* selected for each class in the classifier is done with the other 8-fold cross-validation which includes all the available samples (as indicated in section 3.5.1).

```
> leukemiasClassifier@generalizationError@classificationGenes.num

      ALL AML CLL CML NoL
CV 1:   6   7   1   3  10
CV 2:   3   2   1   8   6
CV 3:   9   2   2   6   5
CV 4:   2  16   2   9  16
CV 5:   3   5   1   8  10
```

3.6 Return III: Gene networks

Together to the classifier and the genes ranking, the third major result that the algorithm *geNetClassifier* produces are the gene networks associated to each class.

The gene networks for each class are built based on association parameters between genes. These association parameters are gene to gene co-expression calculated using a correlation coefficient (*Pearson* by default) and gene to gene interactions derived from *mutual information* (MI) analysis (*mi.empirical* entropy estimator from the R package minet [4]); both calculated along all the samples of each class of the studied dataset.

The *correlations* and *interactions* also allow to find possible redundancy between the genes as features in the classification procedure. Such redundancy can be tested by producing comparative classifiers that include or not the associated genes. Usually, classifiers without redundant genes need less features for classification.

The *...@genesNetwork* slot contains the list of networks.

```
> leukemiasClassifier@genesNetwork

$ALL
Attribute summary of the GenesNetwork:
```

```
Number of nodes (genes): [1] 1027
Number of edges (relationships): [1] 1942

$AML
Attribute summary of the GenesNetwork:
Number of nodes (genes): [1] 400
Number of edges (relationships): [1] 296

$CLL
Attribute summary of the GenesNetwork:
Number of nodes (genes): [1] 1916
Number of edges (relationships): [1] 18506

$CML
Attribute summary of the GenesNetwork:
Number of nodes (genes): [1] 949
Number of edges (relationships): [1] 6540

$NoL
Attribute summary of the GenesNetwork:
Number of nodes (genes): [1] 400
Number of edges (relationships): [1] 1993
```

```
> overview(leukemiasClassifier@genesNetwork$AML)
```

```
getNodes(...)[1:10]:
 [1] "ENSG00000078399" "ENSG00000143995" "ENSG00000185275" "ENSG00000154188"
 [5] "ENSG00000133101" "ENSG00000198795" "ENSG00000106004" "ENSG00000155792"
 [9] "ENSG00000119919" "ENSG00000106236"
... (400 nodes)

getEdges(...)[1:5,]:
     gene1             class1 gene2             class2 relation
[1,] "ENSG00000078399" "AML"  "ENSG00000143995" "AML"  "Correlation - pearson"
[2,] "ENSG00000154188" "AML"  "ENSG00000198795" "AML"  "Correlation - pearson"
[3,] "ENSG00000078399" "AML"  "ENSG00000106004" "AML"  "Correlation - pearson"
[4,] "ENSG00000154188" "AML"  "ENSG00000155792" "AML"  "Correlation - pearson"
[5,] "ENSG00000119919" "AML"  "ENSG00000108511" "AML"  "Correlation - pearson"
     value
[1,] "0.922460476283629"
[2,] "0.804443836092871"
[3,] "0.836149615702043"
[4,] "0.815177435058601"
[5,] "0.940367679337551"
... (296 edges)
```

Each of the networks in this list is an object of the class *GenesNetwork*. This class offers some functions to retrieve and count the edges and nodes, and also to subset the network (*getSubNetwork()*). Note that *getNodes()* includes all possible nodes even if they are no linked by edges.

```
> getNumEdges(leukemiasClassifier@genesNetwork$AML)

[1] 296

> getNumNodes(leukemiasClassifier@genesNetwork$AML)

[1] 400

> getEdges(leukemiasClassifier@genesNetwork$AML)[1:5,]

     gene1             class1 gene2             class2 relation
[1,] "ENSG00000078399" "AML"  "ENSG00000143995" "AML"  "Correlation - pearson"
[2,] "ENSG00000154188" "AML"  "ENSG00000198795" "AML"  "Correlation - pearson"
[3,] "ENSG00000078399" "AML"  "ENSG00000106004" "AML"  "Correlation - pearson"
[4,] "ENSG00000154188" "AML"  "ENSG00000155792" "AML"  "Correlation - pearson"
[5,] "ENSG00000119919" "AML"  "ENSG00000108511" "AML"  "Correlation - pearson"
     value
[1,] "0.922460476283629"
[2,] "0.804443836092871"
[3,] "0.836149615702043"
[4,] "0.815177435058601"
[5,] "0.940367679337551"

> getNodes(leukemiasClassifier@genesNetwork$AML)[1:12]

 [1] "ENSG00000078399" "ENSG00000143995" "ENSG00000185275" "ENSG00000154188"
 [5] "ENSG00000133101" "ENSG00000198795" "ENSG00000106004" "ENSG00000155792"
 [9] "ENSG00000119919" "ENSG00000106236" "ENSG00000148154" "ENSG00000108511"
```

The function *network2txt()* allows to save or export the networks as text files. This function produces two text files: one with the information about the *nodes* and another with the information about the *edges*. They are flat text files (.txt). In the case of the *edges* file, it includes the nodes that interact (gene1 – gene2), the type of link (correlation or interaction) and the value of such relation.

```
> network2txt(leukemiasClassifier@genesNetwork, filePrefix="leukemiasNetwork")
```

To produce just the files with the information about the *edges*:

```
> geneNtwsInfo <- lapply(leukemiasClassifier@genesNetwork,
+      function(x) write.table(getEdges(x),
+      file=paste("leukemiaNtw_",getEdges(x)[1,"class1"],".txt",sep="")))
```

These flat text files allow to export the networks to external software (e.g. *Cytoscape*, http://www.cytoscape.org).

The networks can also be exported using direct R connectors (e.g. RCytoscape) with the igraph objects returned by the function *plotNetwork* (sec. 6.4).

For more information see the class help *?GenesNetwork*.

4 External validation: query with new samples of known class

Once a classifier is built for a group of diseases or disease subtypes, it can be queried with new samples to know their class. However, before proceeding with samples whose class is unknown, an external validation is normally performed. An external validation consists on querying the classifier with several samples whose class is *a priori* known, in order to see if the classification is done correctly. As indicated in section 3.5.2, if the number of known samples is limited (as it is usually the case) to avoid leaving a sub-set of known samples out of the training, *geNetClassifier()* provides the *generalization error* option, which will simulate an external validation by using cross-validation. Despite this possibility, it is clear that using external samples (totally independent to the classifier built) is the best option to validate its performance.

In this section, we will proceed with an example of external validation with the leukemia's classifier. In *leukemiasEset*, the class of all the available samples is known *a priori*. Since we had 60 samples in the initial leukemia dataset and only 50 were used to train the classifier, the 10 remaining can be used for external validation.

The first step is to select the 10 samples that were not used for training:

```
> testSamples <- c(1:60)[-trainSamples]
> testSamples

 [1] 11 12 23 24 35 36 47 48 59 60
```

The classifier is then be asked about the class of these 10 samples using *queryGeNetClassifier()*:

```
> queryResult <- queryGeNetClassifier(leukemiasClassifier,
+ leukemiasEset[,testSamples])
```

This query will return the class that each sample has been assigned to, which will be saved into $class. It also returns the probabilities of assignment of each sample to each class in $probabilities.

```
> queryResult$class

GSM330195.CEL GSM330201.CEL GSM330611.CEL GSM330612.CEL GSM331037.CEL
          ALL           ALL           AML           AML           CLL
GSM331048.CEL GSM331392.CEL GSM331393.CEL GSM331675.CEL GSM331677.CEL
          CLL           CML           CML           NoL           NoL
Levels: ALL AML CLL CML NoL

> queryResult$probabilities

    GSM330195.CEL GSM330201.CEL GSM330611.CEL GSM330612.CEL GSM331037.CEL
ALL    0.82480476    0.72132949    0.04584317    0.03853380    0.04233982
AML    0.04145204    0.05669690    0.68053161    0.84706650    0.09093176
CLL    0.02591494    0.03200663    0.09622283    0.02028114    0.75041107
CML    0.04409894    0.08325862    0.08198307    0.07359096    0.04732196
```

```
NoL    0.06372931    0.10670835    0.09541932    0.02052760    0.06899539
    GSM331048.CEL GSM331392.CEL GSM331393.CEL GSM331675.CEL GSM331677.CEL
ALL    0.04115917    0.04569346    0.02645151    0.05492039    0.02213885
AML    0.09742257    0.17443163    0.02549073    0.05510842    0.04907441
CLL    0.71914364    0.13181179    0.03923288    0.09748276    0.01016039
CML    0.07715866    0.56354463    0.87901701    0.04714128    0.03225649
NoL    0.06511596    0.08451848    0.02980787    0.74534715    0.88636986
```

Since the real class of the samples is known, we can create a confusion matrix. Note: For using this matrix as input in upcoming functions the real classes should be placed as row names (*rownames*) and the predicted classes (assigned by the classifier) as column names (*colnames*).

```
> confusionMatrix <- table(leukemiasEset[,testSamples]$LeukemiaType,
+ queryResult$class)
```

Once we have executed the query, *externalValidation.stats()* can be used to calculate the parameters to evaluate the classifier (Section 3.5.2).

```
> externalValidation.stats(confusionMatrix)
```

```
$byClass
    Sensitivity Specificity MCC CallRate
ALL         100         100 100      100
AML         100         100 100      100
CLL         100         100 100      100
CML         100         100 100      100
NoL         100         100 100      100

$global
       Accuracy CallRate
Global      100      100

$confMatrix
    ALL AML CLL CML NoL NotAssigned
ALL   2   0   0   0   0           0
AML   0   2   0   0   0           0
CLL   0   0   2   0   0           0
CML   0   0   0   2   0           0
NoL   0   0   0   0   2           0
```

The class to class assignment probability matrix, that gives support to the confusion matrix, can be also created for the external validation analysis:

```
> externalValidation.probMatrix(queryResult,
+ leukemiasEset[,testSamples]$LeukemiaType, numDecimals=3)
```

```
      ALL   AML   CLL   CML   NoL
ALL 0.773 0.049 0.029 0.064 0.085
AML 0.042 0.764 0.058 0.078 0.058
CLL 0.042 0.094 0.735 0.062 0.067
CML 0.036 0.100 0.086 0.721 0.057
NoL 0.039 0.052 0.054 0.040 0.816
```

4.1 Assignment conditions

queryGeNetClassifier() includes an expert-like approach to decide if a sample is assigned to a class: instead of directly assigning a sample to the class with the highest probability, it takes into account the probability of belonging to the class and the probability of the closest class before taking the final decision.

By default, the probability to assign a sample to a given class should be at least double than the *random probability*, and the difference with the next likely class should also be higher than 0.8 times the *random probability*. For example, to assign a sample in a 5 class classifier, the highest probability should be at least 40% (2 x 0.20 = 0.40) and the probability of belonging to the closest class should be at least 16% lower than the highest (0.8 x 0.20 = 0.16). This implies that if a sample's probability to belong to one class is 55% and to belong to another class is 40%, since the the difference is lower than 16%, it is not clear enough, and it will be left as a *NotAssigned* (NA). This feature allows modulation of the assignment to resembles expert decision-making.

To allow adapting these conditions, *queryGeNetClassifier()* includes two coefficients that determine the *minimum probability for assignment* (minProbAssignCoeff), and the *minimum difference between the of the first and the second classes* (minDiffAssignCoeff). If these two coefficients are set up to 0 all samples will be assigned to the most likely class and therefore no samples will be left as *NotAssigned*.

```
> queryResult_AssignAll <- queryGeNetClassifier(leukemiasClassifier,
+     leukemiasEset[,testSamples], minProbAssignCoeff=0, minDiffAssignCoeff=0)
> which(queryResult_AssignAll$class=="NotAssigned")

integer(0)
```

On the contrary, the thresholds can be raised to increase the the certainty of the assignments: i.e. by setting the coefficients to 1.5 and 1, the minimum probability to be assigned is 0.6 (**1.5** x 2 x 0.20) and the minimum difference between first and second class probabilities is 0.2 (**1** x 0.20).

```
> queryResult_AssignLess <- queryGeNetClassifier(leukemiasClassifier,
+     leukemiasEset[,testSamples], minProbAssignCoeff=1.5, minDiffAssignCoeff=1)
> queryResult_AssignLess$class

GSM330195.CEL GSM330201.CEL GSM330611.CEL GSM330612.CEL GSM331037.CEL
          ALL           ALL           AML           AML           CLL
GSM331048.CEL GSM331392.CEL GSM331393.CEL GSM331675.CEL GSM331677.CEL
          CLL   NotAssigned           CML           NoL           NoL
Levels: ALL AML CLL CML NoL NotAssigned
```

In this case, these samples were left as *NotAssigned*:

```
> t(queryResult_AssignLess$probabilities[,
+     queryResult_AssignLess$class=="NotAssigned", drop=FALSE])

                     ALL       AML       CLL       CML        NoL
GSM331392.CEL 0.04569346 0.1744316 0.1318118 0.5635446 0.08451848
```

To help understanding how these thresholds behave for a specific dataset, if *geNetClassifier()* is executed with *estimateGError=TRUE*, it generates a plot presenting the assignment probabilities for each sample. This plot shows the probability of the most likely class *versus* the probability difference with next likely class for each sample. Therefore, it allows to view the effects of the 2 coefficients (*minProbAssignCoeff* and *minDiffAssignCoeff*) in the assignment.

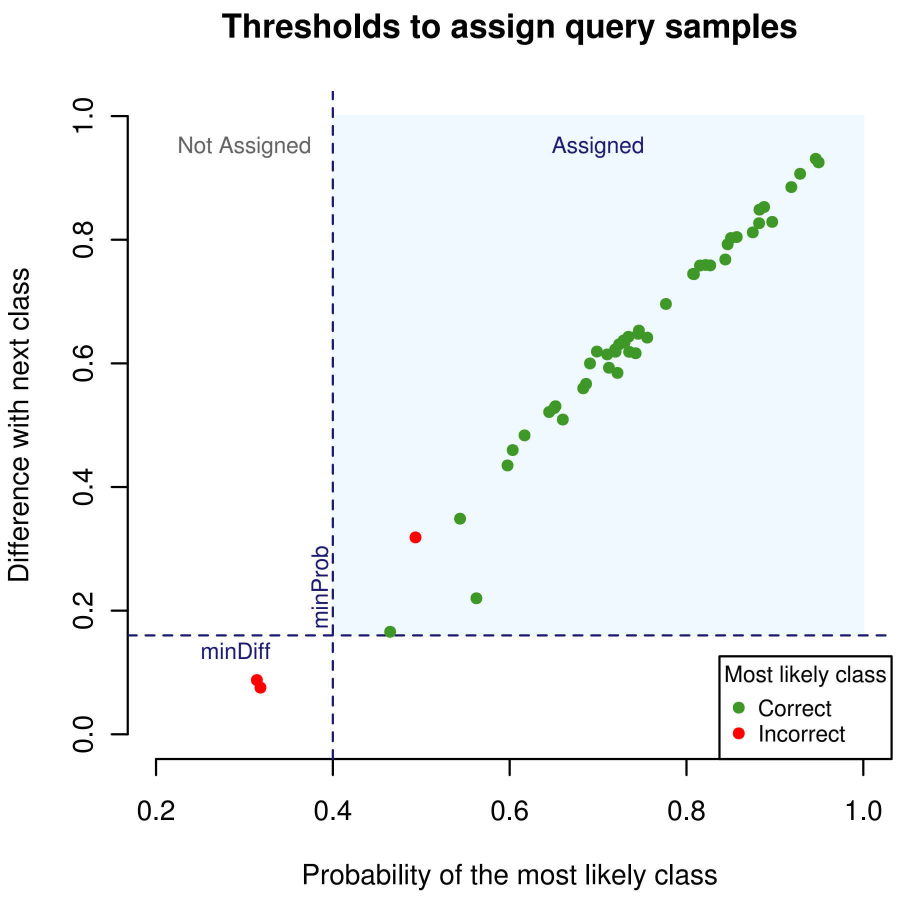

Figure 6. Assignment probabilities plot: It shows for each sample the probability of its most likely class *versus* the difference in probability with the next likely class. **Green** dots indicate that the probability of the most likely class is the correct class. **Red** dots indicate that the probability of the most likely class is not the correct class and, if assigned, such sample would have been missclassified. Dotted lines represent the chosen thresholds. The green area between them shows the samples that are actually assigned, those out of the green area are left as *NotAssigned*.

The plot in Figure 6 was obtained through the execution of *geNetClassifier()* with the leukemia's dataset. It shows that there are several samples under the assignment thresholds: these samples are left as *NotAssigned*. Out of these not assigned samples, the highest probability of some of them was to the real class (green), but some others was to an incorrect class (red). If the classifier had assigned the samples in red, it would have been an incorrect assignment.

5 Sample classification: query with new samples of unknown class

Once a classifier is built for a group of diseases or biological states, we can take external samples from new patients or new studies to query the classifier and know their class type.

Since we had 60 samples in the initial leukemia dataset and only 50 were used in the classifier, the 10 not used for training can be used as new samples to query the classifier and find out their class. In this case we will consider that the class of these samples is unknown.

```
> testSamples <- c(1:60)[-trainSamples]
```

queryGeNetClassifier() can then be used to ask the classifier about the class of the new samples.

```
> queryResult_AsUnkown <- queryGeNetClassifier(leukemiasClassifier,
+ leukemiasEset[,testSamples])
```

In the field *$class* of the return, we can see the class that each sample has been assigned to.

```
> names(queryResult_AsUnkown)

[1] "call"          "class"         "probabilities"

> queryResult_AsUnkown$class

GSM330195.CEL GSM330201.CEL GSM330611.CEL GSM330612.CEL GSM331037.CEL
          ALL           ALL           AML           AML           CLL
GSM331048.CEL GSM331392.CEL GSM331393.CEL GSM331675.CEL GSM331677.CEL
          CLL           CML           CML           NoL           NoL
Levels: ALL AML CLL CML NoL
```

If there were samples that had not been assigned to any class, they would be marked as*NotAssigned*. In the field *$probabilities*, we could see the probability of each sample to belong to each class. All these steps are very similar to the ones describes in section 4.1.

```
> t(queryResult_AsUnkown$probabilities[ ,
+ queryResult$class=="NotAssigned"])

     ALL AML CLL CML NoL
```

The function *querySummary()* provides a summary of the results by counting the number of samples that were assigned to each class and with which probabilities. It is a good way to have an overview of the classification results. In this case, the 100% *call rate* indicates that all samples have been assigned.

```
> querySummary(queryResult_AsUnkown, numDecimals=3)
```

```
$callRate
[1] 100

$assigned
    Count MinProb MaxProb  Mean    SD
ALL     2   0.721   0.825 0.773 0.073
AML     2   0.681   0.847 0.764 0.118
CLL     2   0.719   0.750 0.735 0.022
CML     2   0.564   0.879 0.721 0.223
NoL     2   0.745   0.886 0.816 0.100

$notAssigned
[1] "All samples have been assigned."
```

6 Functions to plot the results

6.1 Plot Ranked Significant Genes: *plot(...@genesRaking)*

As indicated in section 3.4.1, the default plot of a *genesRanking* can be obtained through the plot() function. This plot represents the gene rank obtained for each class *versus* the posterior probability of the genes.

```
> plot(leukemiasClassifier@genesRanking)
```

Some of the parameters to personalize this plot are:

- *lpThreshold* to set the value of the posterior probability threshold (marked as an horizontal line in the plot)
- *numGenesPlot* to determine the maximum number of genes that will be plot

```
> plot(leukemiasClassifier@genesRanking, numGenesPlot=3000,
+ plotTitle="5 classes: ALL, AML, CLL, CML, NoL", lpThreshold=0.80)
```

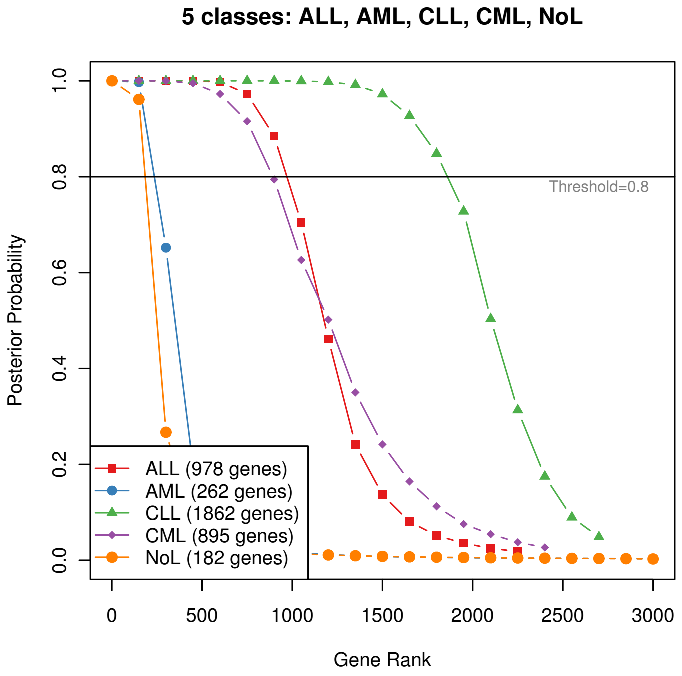

Figure 7. Plot of the posterior probabilities of the genes of 4 leukemia classes and the non-leukemia controls, ordering the genes according to their rank and setting the *lpThreshold* at 0.80.

calculateGenesRanking() allows to calculate (and plot) the ranking for a given data set without building the classifier:

```
> ranking <- calculateGenesRanking(leukemiasEset[,trainSamples],
+ "LeukemiaType")
```

6.2 Plot Gene Expression Profiles: *plotExpressionProfiles()*

The function *plotExpressionProfiles()* generates an overview of the expression profile of each gene along all the samples contained in the studied dataset. The plot will be saved as a PDF if *fileName* is indicated. The parameter *geneLabels* can be used to show a different name to the one included in the expression matrix (i.e. gene symbol instead of ENSEMBL ID or *Affymetrix* ID).

To plot the expression of 4 specific genes across the samples included in the leukemia's set:

```
> data(geneSymbols)
> topGenes <- getRanking(
+ getTopRanking(leukemiasClassifier@classificationGenes,numGenesClass=1),
+ showGeneID=TRUE)$geneID
> plotExpressionProfiles(leukemiasEset, topGenes[,c("ALL","AML"), drop=FALSE],
+     sampleLabels="LeukemiaType", geneLabels=geneSymbols)
```

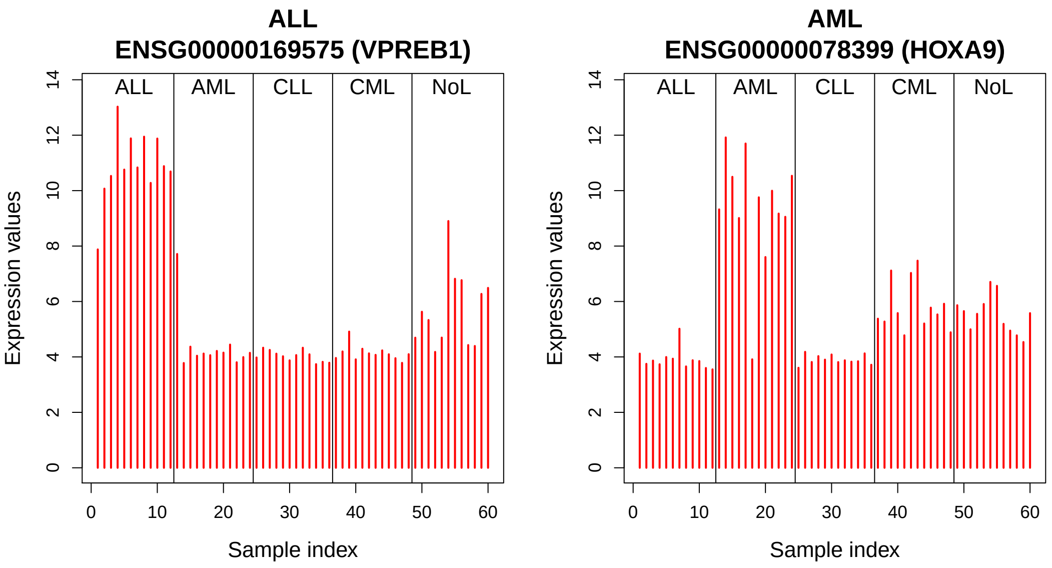

Figure 8. Plot of the expression profiles across 60 samples of 2 genes.

If a *geNetClassifierReturn* object is provided instead of a list of genes, it will plot the expression of all the genes used for training the classifier:

```
> plotExpressionProfiles(leukemiasEset[,trainSamples], leukemiasClassifier,
+     sampleLabels="LeukemiaType", fileName="leukExprs_trainSamples.pdf")
```

To plot the expression of all the genes chosen for classification for a specific class, for example AML:

```
> classGenes <- getRanking(leukemiasClassifier@classificationGenes,
+     showGeneID=TRUE)$geneID[,"AML"]
> plotExpressionProfiles(leukemiasEset, genes=classGenes,
+     sampleLabels="LeukemiaType", geneLabels=geneSymbols, fileName="AML_genes.pdf")
```

These plots can be modified in several ways, for example coloring specific samples or classes, or plotting the expression as boxplot

- Coloring specific samples or classes:

```
> plotExpressionProfiles(leukemiasEset, genes=topGenes[,3, drop=FALSE],
+                        sampleLabels="LeukemiaType",
+                        showMean=TRUE, identify=FALSE,
+                        sampleColors=c("grey","red")
+                        [(sampleNames(leukemiasEset)%in% c("GSM331386.CEL","GSM3313
```

```
> plotExpressionProfiles(leukemiasEset, genes=topGenes[,3, drop=FALSE],
+                        sampleLabels="LeukemiaType",
+                        showMean=TRUE, identify=FALSE,
+                        classColors=c("red","red", "blue","red","red"))
```

- Plotting the expression as boxplot (grouped by classes):

```
> plotExpressionProfiles(leukemiasEset, genes=topGenes[,3, drop=FALSE],
+                        sampleLabels="LeukemiaType",
+                        type="boxplot", geneLabels=geneSymbols, sameScale=FALSE)
```

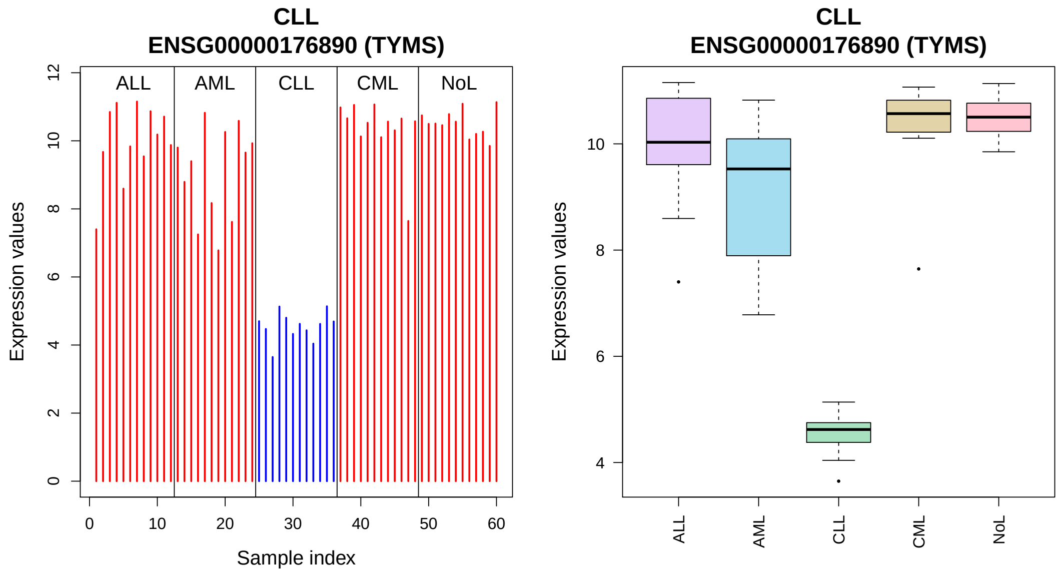

Figure 9. Two different versions of expression plot.

See *?plotExpressionProfiles* for more details.

6.3 Plot Genes Discriminant Power: *plotDiscriminantPower()*

The *discriminant power* is a parameter derived from the classifier's *support vectors* which resembles the power of each gene to mark the difference between classes.

The multi-class SVM algorithm (One-versus-One, OvO) produces a set of *support vectors* for each binary comparison between classes. Such *support vectors* include the *Lagrange coefficients* (alpha) for all the genes selected for the classification. Therefore, we can assign to each gene the sum of the *Lagrange coefficients* of all the *support vectors* of each class (represented as piled up bars in the plot). The *discriminant power* is then calculated as the difference between the value of the largest class and the closest (the distance marked by two red lines in the plot). In conclusion, the *discriminant power* is a parameter that allows the characterization of the genes based in their capacity to separate different classes (i.e. different diseases or diseases subtypes compared).

The *discriminant power* is calculated for each gene included in the classifier (the *@classificationGenes*) when it is built *geNetClassifier()*). The *plotDiscriminantPower()* function is included in the package to generate a graphic representation of the *discriminant power*.

```
> plotDiscriminantPower(leukemiasClassifier,
+ classificationGenes="ENSG00000169575")
```

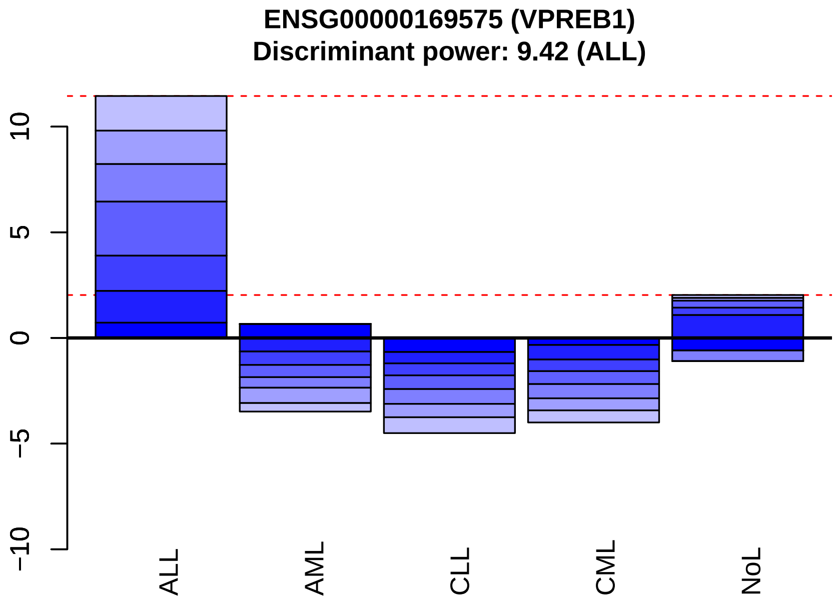

Figure 10. Plot of the discriminant power of gene VPREB1 (ENSG00000169575). The plot shows that this gene identifies class ALL and the closest class is NoL.

The next example shows the discriminant power of the top genes of a class. In order to plot more than 20 genes, or to save the plots as PDF, provide a *fileName*.

```
> discPowerTable <- plotDiscriminantPower(leukemiasClassifier,
+ classificationGenes=getRanking(leukemiasClassifier@classificationGenes,
+ showGeneID=TRUE)$geneID[1:4,"AML",drop=FALSE], returnTable=TRUE)
```

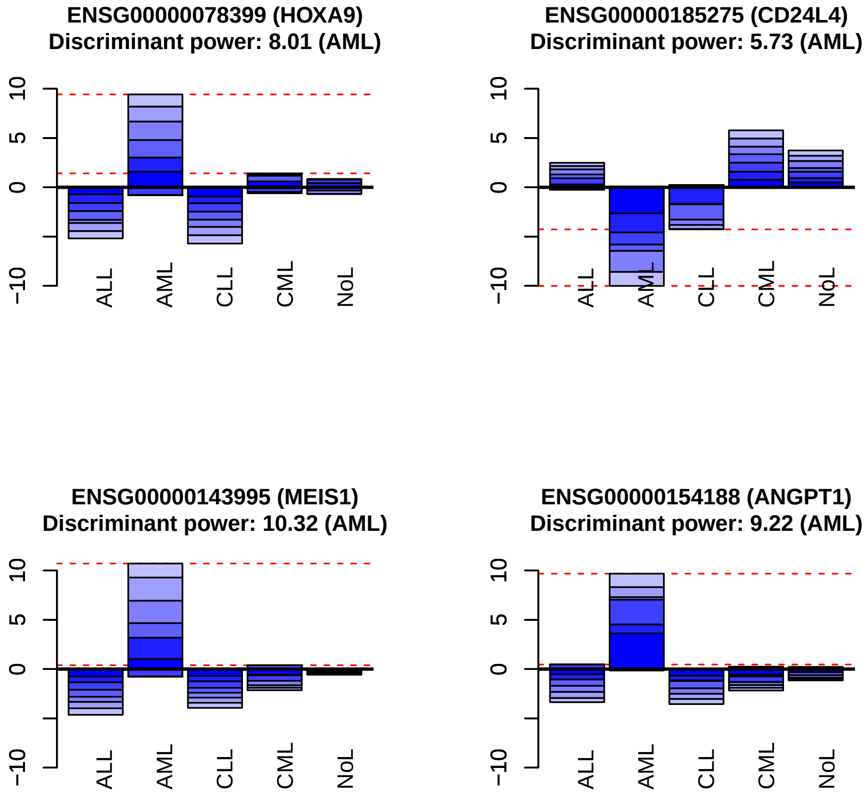

Figure 11. Plot of the discriminant power of the 4 genes that best discriminate AML class from the other classes. The figures indicate that MEIS1 (ENSG00000143995) presents the highest discriminant power. This gene encodes a homeobox protein that has been involved in myeloid leukemia. A high discriminant power can help to identify gene markers.

Some of the options to personalize the plot are *classNames* to provide a different name for the classes and textitgeneLabels to provide a alias for the genes. As usual, more details about the function are available at *?plotDiscriminantPower*.

6.4 Plot Gene Networks: *plotNetwork()*

The package also includes some functions to manipulate the networks produced by *geNetClassifier()* (i.e. select part of a network and personalize the plots).

Step 1: Select a network or sub-network.
getSubNetwork() allows to select sub-networks. i.e. the sub-network containing only the classification genes:

```
> clGenesSubNet <- getSubNetwork(leukemiasClassifier@genesNetwork,
+ leukemiasClassifier@classificationGenes)
```

Step 2: Get the info of the genes to plot.
genesDetails() provides the available information about the genes. This information can be shown in the network: The gene name will be the node label. The expression of the gene will be shown with the node color, and the discriminant power will determine its size. In case the network includes genes selected for classification and genes which were not selected, the genes selected for classification will be plot as squares and the not selected as circles (only available for PDF plot, not on the dynamic view). For more details see the network legend in figure 14.

```
> clGenesInfo <- genesDetails(leukemiasClassifier@classificationGenes)
```

Step 3: Plot the network.
The network plots can be produced either using R interactive view (*tkplot* from *igraph*) or plotted as saved PDF files. Use *plotType="pdf"* to save the network as a static PDF file. This option is recommended to produce an overview of several networks. To produce interactive networks skip this argument. Iteractive plotscan be exported as a *postscript* files (.eps).

Some plot examples:
Network of ALL classification genes:

```
> plotNetwork(genesNetwork=clGenesSubNet$ALL, genesInfo=clGenesInfo)
```

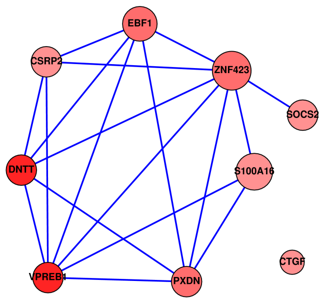

Figure 12. Gene network obtained for class ALL including the 9 classification genes selected for this disease.

Only connected nodes from ALL classification genes network:

```
> plotNetwork(genesNetwork=clGenesSubNet$ALL, genesInfo=clGenesInfo,
+ plotAllNodesNetwork=FALSE, plotOnlyConnectedNodesNetwork=TRUE)
```

AML network of the top 30 genes from the ranking (calculated as co-expression and mutual information):

```
> top30g <- getRanking(leukemiasClassifier@genesRanking,
+ showGeneID=TRUE)$geneID[1:30,]
> top30gSubNet <- getSubNetwork(leukemiasClassifier@genesNetwork, top30g)
> top30gInfo <- lapply(genesDetails(leukemiasClassifier@genesRanking),
+ function(x) x[1:30,])
> plotNetwork(genesNetwork=top30gSubNet$AML, genesInfo=top30gInfo$AML)
```

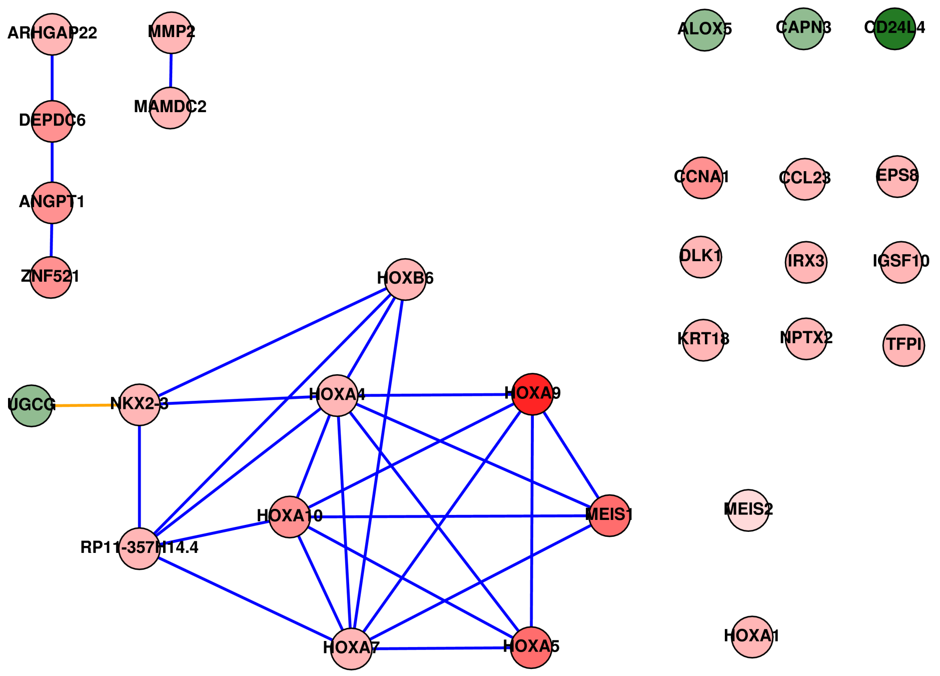

Figure 13. Gene network obtained for class AML including the top 30 genes selected from the gene ranking of this disease.

Network of the top 100 genes from AML ranking.
A preview of this network is automatically plotted for every class by *geNetClassifier()* if *plotsName* is provided.

```
> top100gRanking <- getTopRanking(leukemiasClassifier@genesRanking,
+ numGenes=100)
> top100gSubNet <- getSubNetwork(leukemiasClassifier@genesNetwork,
+ getRanking(top100gRanking, showGeneID=TRUE)$geneID)
> plotNetwork(genesNetwork=top100gSubNet,
+ classificationGenes=leukemiasClassifier@classificationGenes,
+ genesRanking=top100gRanking, plotAllNodesNetwork=TRUE,
+ plotOnlyConnectedNodesNetwork=TRUE, labelSize=0.4,
+ plotType="pdf", fileName="leukemiasNetwork")
```

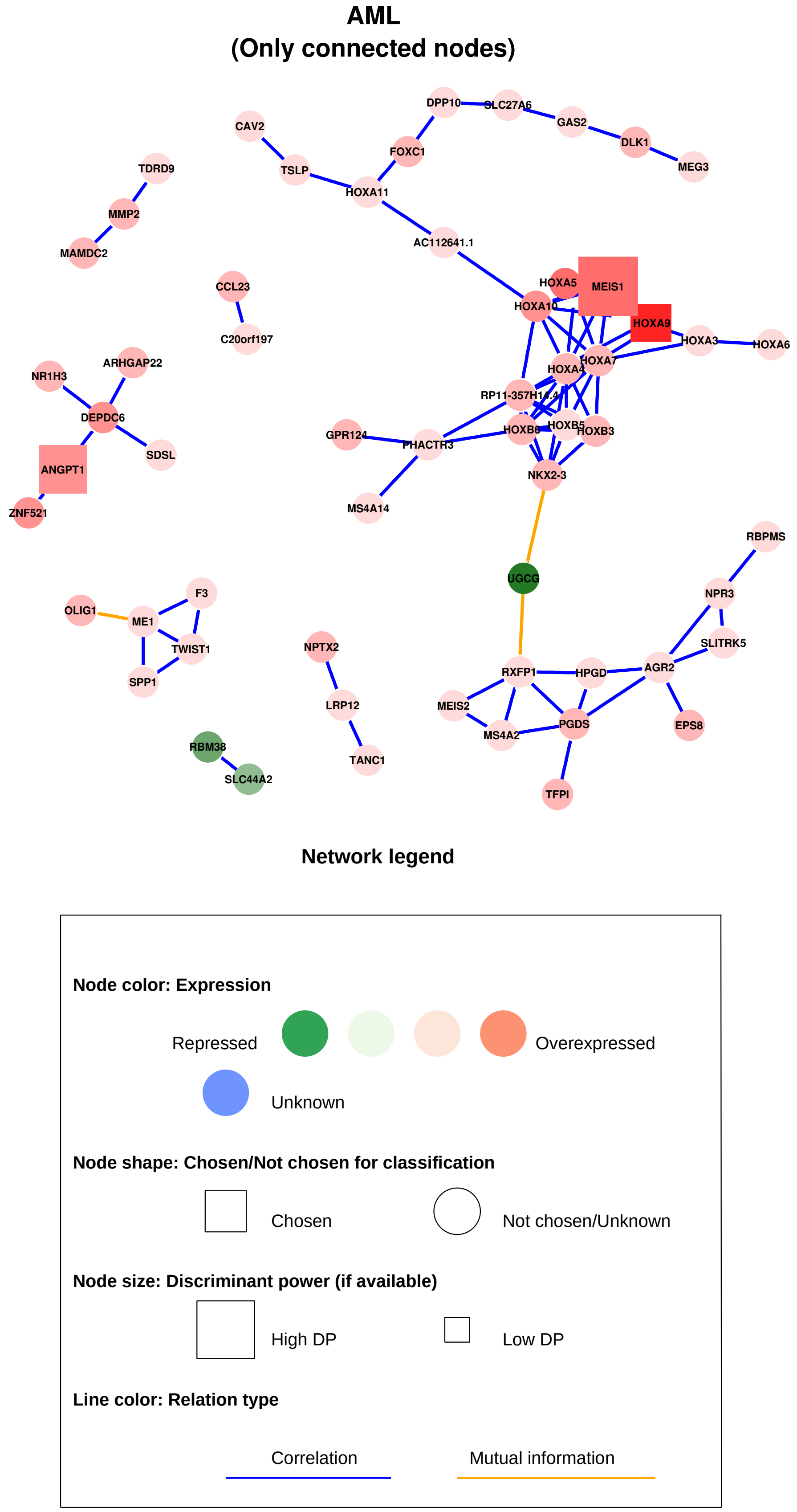

Figure 14. Gene network obtained for class AML selecting the 100 top genes from the gene ranking of this disease, but presenting only the connected nodes. The figure also includes the network legend indicating the meaning of the shapes and colors given to the nodes and edges.

Acknowledgements

This work was supported by Instituto de Salud Carlos III and by a grant from the Junta de Castilla y Leon and the European Social Fund to S.A and C.D.

References

[1] Haferlach T, Kohlmann A, Wieczorek L, Basso G, Kronnie GT, Bene MC, De Vos J, Hernandez JM, Hofmann WK, Mills KI, Gilkes A, Chiaretti S, Shurtleff SA, Kipps TJ, Rassenti LZ, Yeoh AE, Papenhausen PR, Liu WM, Williams PM, Foa R (2010). *Clinical utility of microarray-based gene expression profiling in the diagnosis and subclassification of leukemia: report from the International Microarray Innovations in Leukemia Study Group.* J Clin Oncol. 28: 2529-2537.

[2] Barrier A, Lemoine A, Boelle PY, Tse C, Brault D, Chiappini F, Breittschneider J, Lacaine F, Houry S, Huguier M, Van der Laan MJ, Speed T, Debuire B, Flahault A, Dudoit S (2005) *Colon cancer prognosis prediction by gene expression profiling.* Oncogene. 24: 6155-6164.

[3] Meyer D, Leischa F, Hornik K (2005). *The supportvector machine under test.* Neurocomputing. 55: 169-186

[4] Meyer PE, Lafitte F, Bontempi G (2008). *minet: A R/Bioconductor package for inferring large transcriptional networks using mutual information.* BMC Bioinformatics. 9: 461.

[5] Risueno A, Fontanillo C, Dinger ME, De Las Rivas J (2010). *GATExplorer: genomic and transcriptomic explorer; mapping expression probes to gene loci, transcripts, exons and ncRNAs.* BMC Bioinformatics. 11: 221.

[6] Morris C (1983). *Parametric empirical Bayes inference: theory and applications.* JASA. 78: 47-65.

[7] Kendziorski CM, Newton MA, Lan H, Gould MN (2003). *On parametric empirical Bayes methods for comparing multiple groups using replicated gene expression profiles.* Statistics in Medicine 22: 3899-3914.

[8] Benjamini Y, Hochberg Y (1995). *Controlling the false discovery rate: A practical and powerful approach to multiple testing.* J. Roy. Statist. Soc. Ser. B. 57: 289-300.
